# Application of operating scenarios and analysis of unstable flow characteristics at various angles of inlet guide vane

**DOI:** 10.1038/s41598-024-73436-2

**Published:** 2024-09-30

**Authors:** Yong-Jin Son, Hyeon-Mo Yang, Kyoung-Yong Lee, Joon Yong Yoon, Young-Seok Choi

**Affiliations:** 1 Industrial Energy R&D Department, Research Institute of Sustainable Development Technology, Korea Institute of Industrial Technology, 89 Yangdaegiro-gil, Ipjang-myeon, Seobuk-gu, Cheonan-si, 31056 Chungcheongnam-do Republic of Korea; 2 Department of Mechanical Engineering, Hanyang University, Seoul, 133-791 Republic of Korea; 3 Mechanical Engineering, Korea University of Technology and Education, 1600, Chungjeol-ro, Byeongcheon-myeon, Dongnam-gu, Cheonan-si, Chungcheongnam-do Republic of Korea; 4 Industrial Technology (Green Processes and Energy System Engineering), University of Science and Technology, 217, Gajeong-ro, Yuseong-gu, Daejeon, 34113 Republic of Korea

**Keywords:** Axial pump, Inlet guide vane, Flow angle, Hydrodynamic performance, Operational scenario, Energy saving, Engineering, Mechanical engineering, Fluid dynamics

## Abstract

In this study, we analyzed the performance characteristics of an axial flow pump with different angles of internally installed inlet guide vanes (IGVs). We predicted the pump’s performance based on changes in the IGV angle and analyzed the impact of these angle variations on pump operation in the low-flow region. Additionally, we used real operational data from two sewage treatment plants to propose efficient operational scenarios. For turbulence flow analysis, the Reynolds-averaged Navier–Stokes equations were discretized based on the finite volume method. The grid formation was evaluated using the grid convergence index to select the optimal grid. Then, the internal flow was analyzed in detail through transient-state analysis. Through fast Fourier transform analysis, we confirmed that adjusting the IGV angle during pump operation in the low-flow region in response to load changes results in more stable operation compared with the existing method (valve control). Overall, our findings verified that energy reduction and efficient operation can be achieved through IGV angle adjustment compared with valve control.

## Introduction

Large volumes of fluid are typically transported in sewage treatment plants. Axial flow pumps are commonly used in such environments because they are suitable for high-capacity fluid transfer. They are suitable for transporting large volumes of fluid due to their ability to operate at high flow rates, particularly under low-head conditions. They are also more convenient than other pumps because their compact design facilitates easy installation and maintenance on-site. One of the components of an axial flow pump, the impeller, is designed to transfer large volumes of fluid. Therefore, the power consumption of an axial flow pump is considerably higher than that of other pumps. Therefore, efficient operational scenarios should be established to reduce power consumption and achieve energy savings and environmental conservation^1^.

The main internal components of an axial flow pump is a bell-mouth-shaped inlet casing, an inlet guide vane (IGV), an impeller (with propeller-type blades), and diffuser vanes. The impeller, which significantly influences the hydraulic performance of the pump, was analyzed for design optimization based on factors such as the rotation angle and tip clearance^2–4^. Furthermore, extensive research has been conducted to analyze internal fluid dynamics and pressure pulsations to enhance pump efficiency and performance^5–9^. Kan et al. improved the performance of Pumps as Turbines (PATs) through design optimization. Specifically, they enhanced cavitation performance by optimizing geometric design parameters. Additionally, they analyzed the impact of tip leakage flow within the pump on overall performance^10–13^. The IGV is a widely used part of turbomachinery. It can help reduce the turbulent flow that may arise in the flow entering the pump before reaching the impeller, thereby introducing a stable flow into the impeller under low-flow conditions. Therefore, before the installation of an IGV, its effect on pump performance should be predicted by adjusting the incoming flow angle into the pump^14,15^. Predicting the effects of the IGV before fabrication can minimize economic losses and trial-and-error processes during manufacturing by reducing the design changes and process adjustments that may arise in production^16,17^.

As the IGV is typically installed in front of the impeller, adjusting the IGV angle enables control of the inflow direction of the fluid entering the impeller. Therefore, the presence or absence of an internal IGV in a pump affects pump performance^18^. Jiao et al.^19^ analyzed the hydraulic performance characteristics and internal pressure pulsations of a pump based on the presence or absence of an IGV. Liu et al.^20^ designed 2D and 3D geometries for IGVs using numerical and experimental methods to analyze the hydraulic performance characteristics and internal pressure pulsations, progressing toward their optimal design. Therefore, to control the fluid flow optimally, Hou et al.^21^ analyzed the efficiency and performance characteristics of a pump based on the number of IGV blades when installed in front of the impeller to determine the optimal number of blades. Zhao et al.^22^ selected the optimal IGV angle by analyzing the internal performance characteristics of a pump at various IGV angles at a design flow rate. Kim et al.^23,24^ analyzed the performance characteristics variation of a pump based on changes in IGV and impeller angles and used this information to determine the optimal angles. Yuchuan et al.^25^ analyzed the impact of IGV angle variations on internal flow and pressure pulsations at a design flow rate. Through this analysis, they confirmed that appropriate changes in the IGV angle influence the unstable flow occurring within the pump. Ahmed et al.^26^ selected the optimal angle for IGV adjustments based on internal flow analysis and cavitation reduction resulting from changes in the IGV angle. IGV installation inside a pump affects the pump performance characteristics and the formation process of internal cavitation^27–30^. The IGV influences the angle of the flow entering the pump, leading to changes in pump performance characteristics^31,32^. Additionally, axial flow pumps inherently undergo hydrodynamic instability in the low-flow region due to the surging caused by changes in the slope of the total head performance curve. Although operation in the low-flow region should be avoided during the use of an axial flow pump, using the characteristics of the IGV to influence the angle of the flow entering the pump can enhance efficiency and expand the pump operation range, improving the efficiency^33,34^.

Compared with a fixed-angle IGV, a variable-angle IGV within a pump allows for more efficient pump operation in response to changes in incoming flow^35^. Feng et al.^36^ analyzed the effects of using a variable-angle IGV and the IGV tip clearance on pump performance characteristics under different loads entering the pump. Kim et al.^37^ analyzed the effects of the thickness of an internally installed variable-angle IGV and diffuser vanes on internal flow and performance characteristics and then selected the optimal thickness. Qian et al.^38^ experimentally confirmed that pump performance and efficiency improve when variable-angle IGVs are installed internally. On this basis, they used numerical methods to predict effective operational strategies by installing a variable-angle IGV in a hydraulic turbine. Other previous studies have consistently demonstrated the effectiveness of using variable-angle IGVs instead of fixed-angle IGVs in pump operation^39,40^.

The preceding studies suggest that the flow angle is altered via the characteristics of an IGV to change the internal flow characteristics of the pump. Optimizing the IGV angle can enhance pump performance and efficiency. However, research is limited on the effect of changing the flow angle using the IGV characteristics in unstable-flow regions, particularly the low-flow region, when operating axial flow pumps in site operation scenarios. Furthermore, the impact of the presence or absence of internally installed variable-angle IGVs in pumps operating in fields on power consumption has yet to be analyzed. Therefore, in this study, we analyzed the impact of altering the flow angle using a variable-angle IGV in the low-flow region of an axial flow pump on internal flow. We also evaluated flow stability through pressure pulsation analysis. Additionally, using real operational data from sewage treatment plants, we predicted operational scenarios based on the presence or absence of flow angle adjustments using a variable-angle IGV. We then analyzed the influence of IGV angle changes on pump shaft power consumption. This study is structured into sections including introduction, design specifications and experimental methods, numerical analysis setup, results, and conclusions. In the section on Design Specifications and Experimental Methods, the introduction of the axial flow pump model and the performance testing methods were discussed. The Numerical Analysis Setup section covered the numerical analysis methods, applied conditions, and validation of the numerical analysis model. The Results section presented an analysis of the internal flow field in response to changes in IGV angles and the application results of the pump operation scenarios. Lastly, the Conclusions section summarized the study and presented the conclusions.

## Design specifications and experiment methods

### Axial pump

**Fig. 1:**
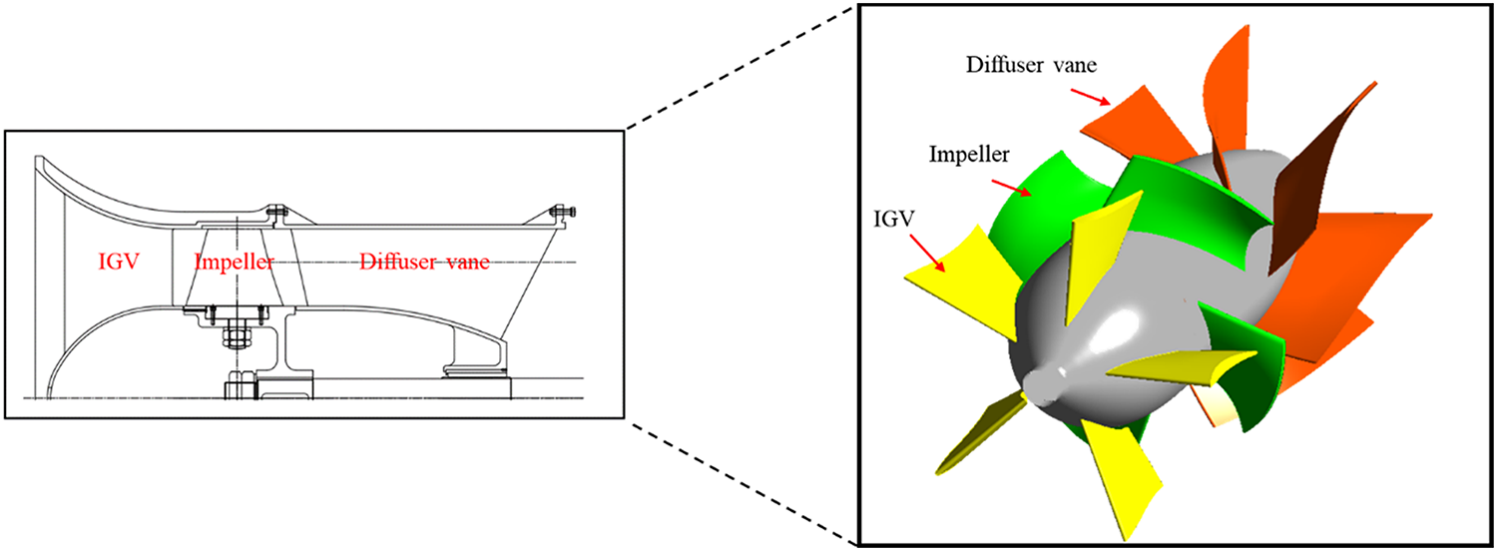
Meridional-view diagram and 3D model of studied axial flow pump (IGV: inlet guide vane).

**Fig. 2:**
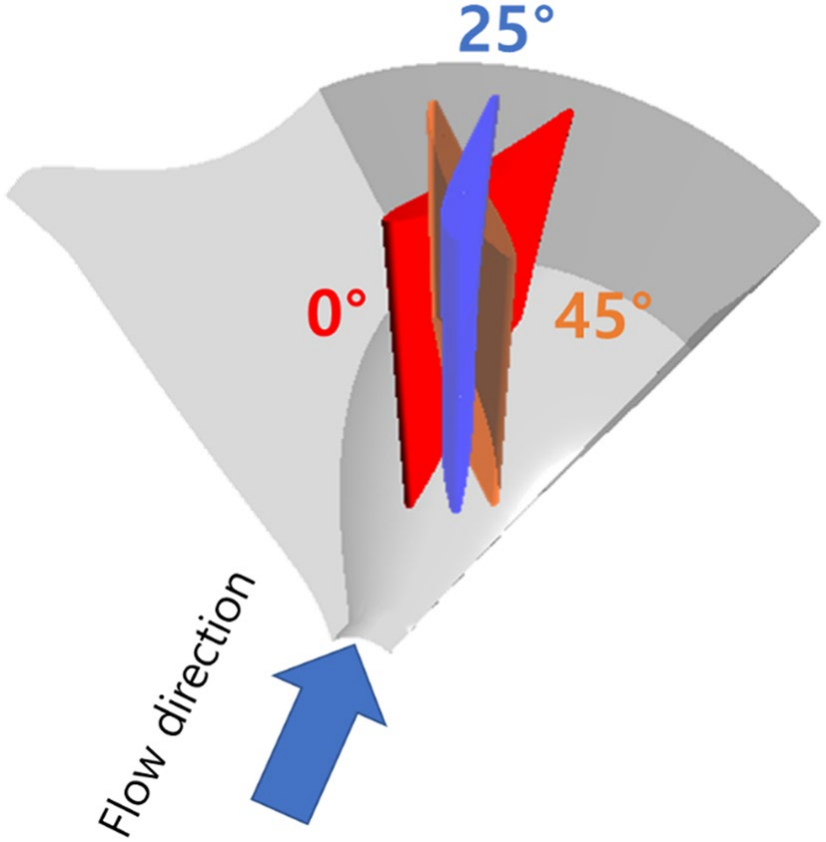
IGV blade angle setup of axial flow pump model.

The components of the studied axial flow pump include a bell-mouth-shaped inlet section, a variable-angle IGV, an impeller, and diffuser vanes. The variable-angle IGV reduces the turbulent flow generated in the flow entering the pump. The IGV influences the incidence angle of the flow entering the impeller, thereby inducing changes in pump performance characteristics. The impeller increases the pressure of the incoming fluid, and the diffuser vanes restore the static pressure. A meridional-plane image and a 3D model of the studied axial flow pump are illustrated in Fig. 1. The variable-angle IGV inside the pump rotates in the impeller’s rotational direction at 0°, 25°, and 45°, as shown in Fig. 2. Changes in the IGV angle modify the incidence angle of the flow entering the impeller. The detailed design specifications of the axial flow pump are presented in Table 1, and the specific speed is non-dimensionalized using Eq. (1). The flow, total head, and shaft power coefficients are non-dimensionalized using Eqs. (2), (3), and (4), respectively.

1 $$\:{N}_{s}=\:\frac{\omega\:\sqrt{Q}}{{\left(gH\right)}^{3/4}}$$

2 $$\:\varPhi\:=\:\frac{{c}_{m2}}{{u}_{2}}$$

3 $$\:\varPsi\:=\:\frac{2gH}{{u}_{2}^{2}}$$

4 $$\:\lambda\:=\:\frac{L}{\frac{1}{2}\rho\:{A}_{2}{u}^{{3}_{2}}}$$

$\:Q$ (m^3^/s), $\:H$ (m), and $\:L$ (W) are the flow rate, total head, and shaft power, respectively. $\:{u}_{2}$ (m/s), $\:{C}_{m2}$ (m/s), $\:\rho\:$ (kg/m^3^), $\:g$ (m/s^2^), and $\:\omega\:$ (rad/s) are the impeller outlet rotational velocity, meridional component of absolute velocity at the impeller outlet, density, gravitational acceleration, and angular velocity, respectively.

**Table 1:** Design specifications of studied axial flow pump.

Description	Value
Specific speed ($\:{N}_{s}$)	2.94
Flow coefficient ($\:\varPhi\:$)	0.242
Total head coefficient ($\:\varPsi\:$)	0.341
Shaft power coefficient ($\:\lambda\:$)	0.108
Rotational speed (rpm)	2,560
Impeller dimeter (mm)	185
Number of de-swirlers	5
Number of impeller blades	4
Number of diffuser vanes	7

### Experimental setup

**Fig. 3:**
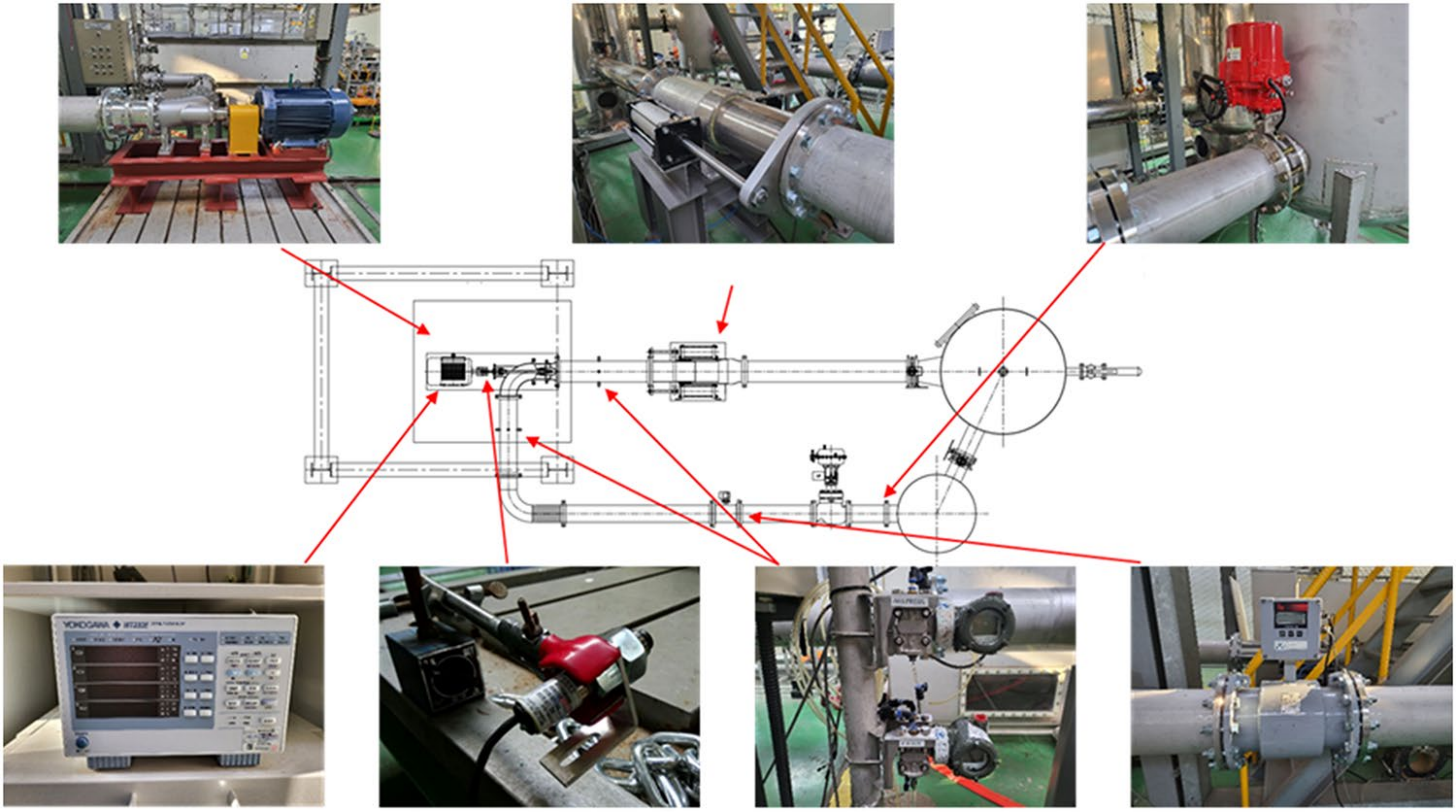
Schematic diagram of axial flow pump test facility and measurement setup.

Experiments on the studied axial flow pump were conducted at the Korea Institute of Industrial Technology (KITECH). Figure 3 shows a schematic diagram of the test equipment for performance evaluation and operation scenario prediction of the axial flow pump, along with the measurement devices. The experiments were conducted in a closed-loop test system equipped with a variable-angle IGV, an impeller, and diffuser vanes. As seen in the diagram, the test equipment and measurement devices included the axial flow pump, a butterfly valve, a power meter, a tachometer, pressure sensors, and an electronic flow meter. The test scope and precision of the measurement items and instruments satisfied the requirements of the KS B6301 standard. For performance evaluation, performance curves were obtained, including design flow points and off-design points, at IGV angles of 0°, 25°, and 45°. Pump operation scenarios through changes in IGV angles were predicted based on actual operational data from sewage treatment plants S and H in Korea.

## Numerical analysis setup

### Computational model and boundary conditions

**Fig. 4:**
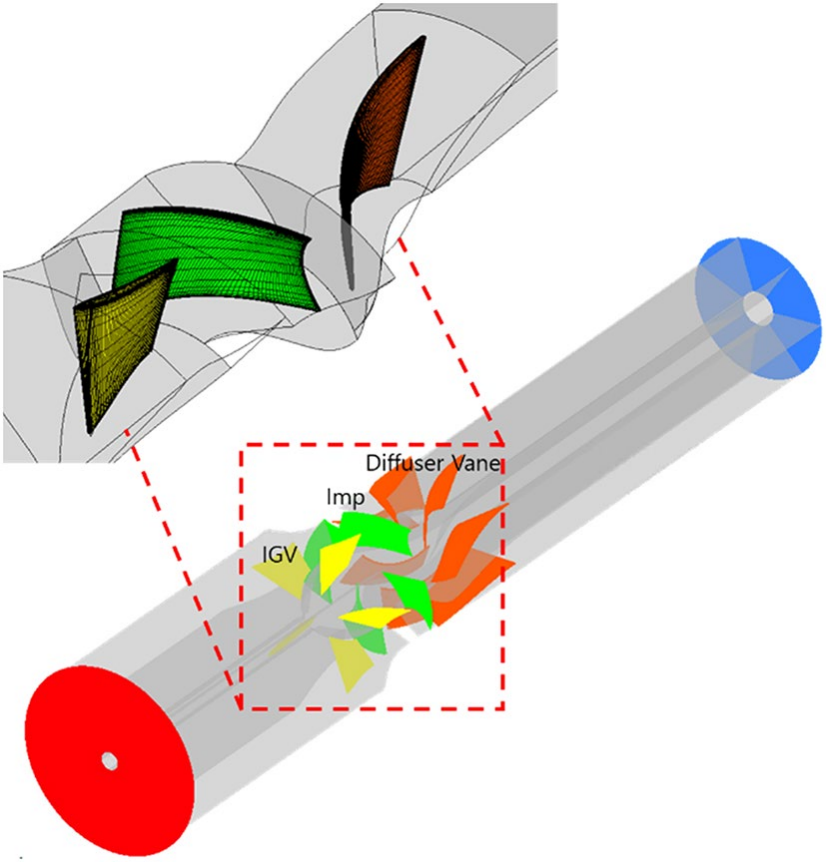
Numerical analysis model and grid system (Imp: impeller).

**Fig. 5:**
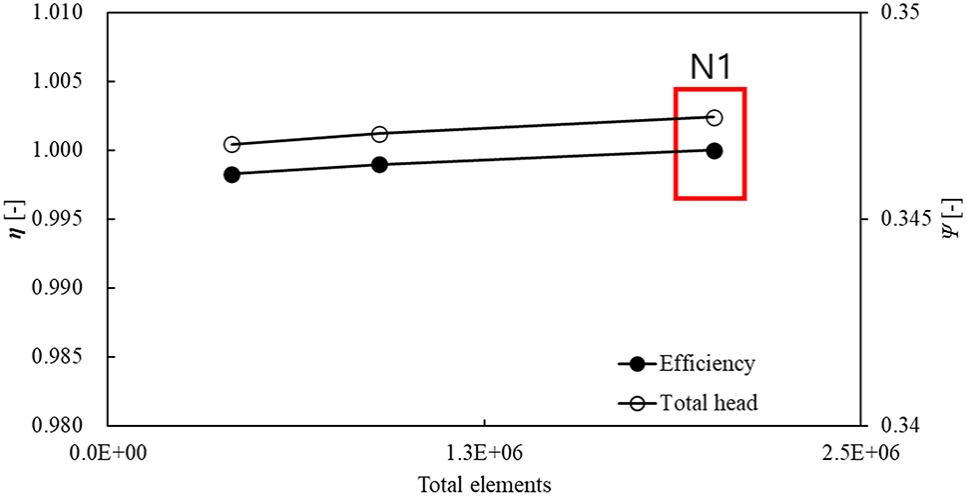
Grid convergence index validation result of numerical analysis model ($\:\eta\:$: normalized total efficiency; $\:\psi\:$: total head coefficient).

The numerical analysis model and grid system used in this study are shown in Fig. 4. The numerical analysis model consisted of a bell-mouth-shaped inlet section, a variable-angle IGV, an impeller, and diffuser vanes, arranged from left to right in the flow direction. The rotating part was limited to the impeller region. The IGV of an axial flow pump has tip clearance. The tip clearances for the studied IGV were as follows: 0.092$\:D$ for the shroud leading-edge tip, 0.006$\:D$ for the shroud trailing-edge tip, 0.015$\:D$ for the hub leading-edge tip, and 0.009$\:D$ for the hub trailing-edge tip. The tip clearance of the leading and trailing edges of the impeller shroud was 0.002$\:D$. For analysis convergence, the inlet section was extended to have the same diameter as the inlet of the variable-angle IGV. The grid system was composed of a hexahedral grid, and it was validated using the grid convergence index (GCI)^41^. The validation results of the grid system are in Fig. 5, and detailed data are shown in Table 2. At the design flow rate, numerical analysis was conducted to compare discretization errors for total head and efficiency across three observed grids. Efficiency was normalized based on the numerical analysis results obtained from the grid corresponding to $\:{N}_{1}$. The GCI_fine_^21^ value for the optimal grid ($\:{N}_{1}$), with approximately $\:2.0\times\:{10}^{7}$ cells, was approximately 0.0029. Because this value met previously proposed convergence criteria^41^, the grid corresponding to $\:{N}_{1}$ was utilized. The numerical analysis was conducted using the commercial computational fluid dynamics (CFD) analysis software Ansys CFX 19.2^42^. For turbulence analysis, the Reynolds-averaged Navier–Stokes equations were discretized based on the finite volume method. The wall surfaces were assigned with no-slip and automatic wall functions. The terms for time variation were added to each governing equation in transient-state analysis. For turbulence modeling, the shear stress transport model^43^ was applied to capture flow separation phenomena accurately. Atmospheric pressure and mass flow rate conditions were imposed in the inlet and outlet sections, respectively. During the transient-state analysis, one rotation of the pump was equivalent to 0.023 s, and data were collected at every 3° for detailed information. A 32-core dual-processor Xeon (2.8 GHz) central processing unit was utilized during the numerical analysis. The computation time for steady-state single-passage analysis was approximately 6 h, and the analysis times for the steady- and transient-state analyses of the entire passage required approximately 13 h and 26 days, respectively.

**Table 2:** Calculation of discretization error for axial pump model.

	$\:{\varnothing}=\text{Efficiency}$	$\:\text{H}=\text{T}\text{o}\text{t}\text{a}\text{l}\:\text{h}\text{e}\text{a}\text{d}$
$\:{N}_{1}$, $\:{N}_{2}$, $\:{N}_{3}$	2.0 × 10^6^, 9.0 × 10^5^, 4.5 × 10^5^	
*r*_*21*_	1.31	
*r*_*32*_	1.30	
*Ø*_*1*_	1	1
*Ø*_*2*_	0.9989	0.9976
*Ø*_*2*_	0.9992	0.9984
GCI_fine_^21^	0.0029	0.0020

### ΦEvaluation of numerical model of axial flow pump

**Fig. 6:**
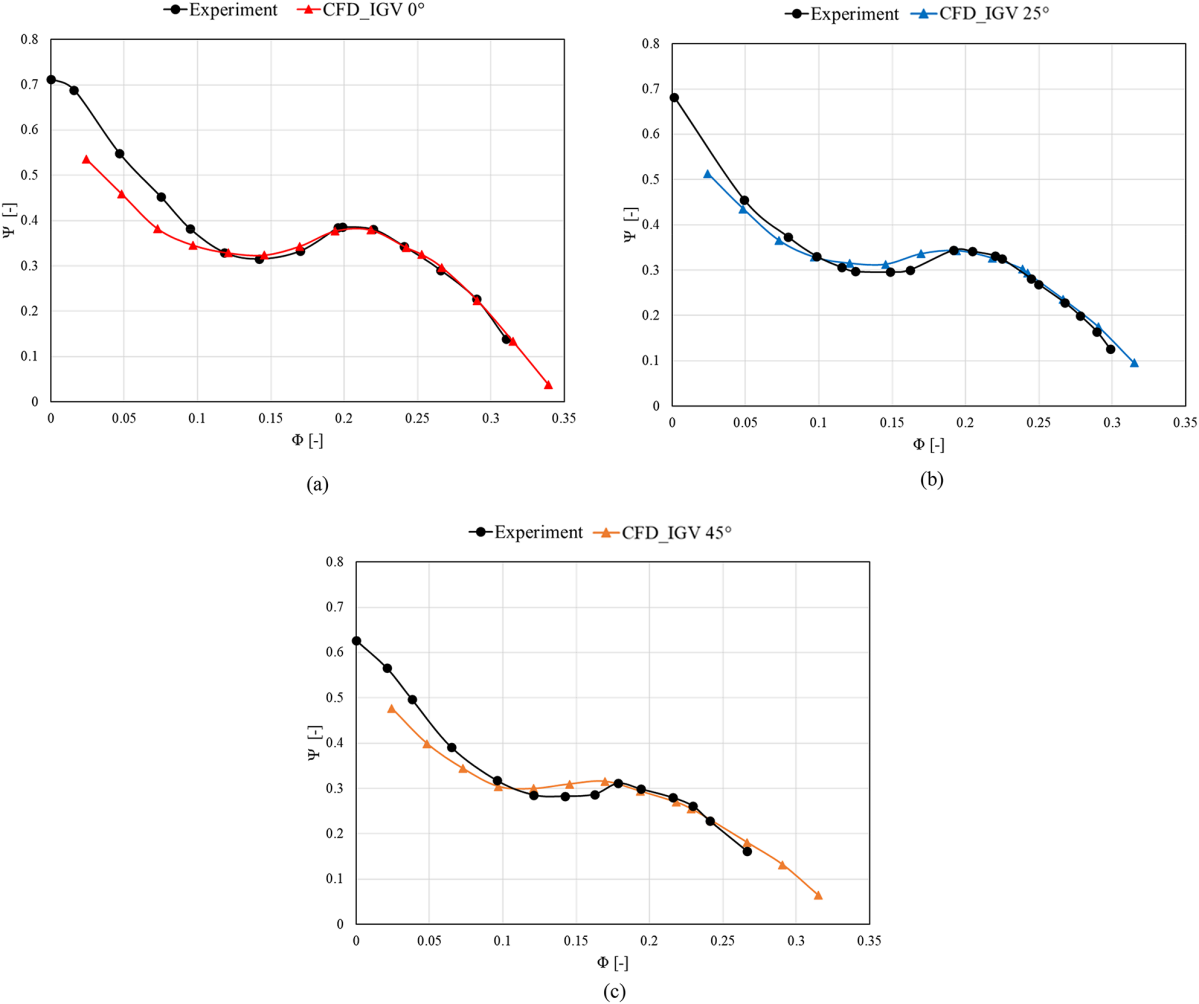
Comparison of numerical analysis and experimental results for model validation (Φ: flow coefficient). (**a**)  IGV angle 0°, (**b**)  IGV angle 25° and (**c**)  IGV angle 45°.

Figure 6 is a comparison of the experimental and numerical analysis results for model validation. The analysis was performed at IGV angles of 0°, 25°, and 45°. The experiment was repeated under the same conditions except for changing the IGV angles to 0°, 25°, and 45°. At the IGV angle of 0°, the same turning points are achieved, indicating similar overall trends. At the IGV angle of 25°, a slight difference is observed relative to the 0° curve in the saddle region, with a maximum difference of approximately 0.8%. Similarly, for the IGV angle of 45°, a maximum difference of approximately 0.9% is seen in the saddle region. This is due to the system resistance caused by the surface roughness of the flow passage used in the experiments and various other factors, resulting in lower predictions compared with the numerical analysis findings. The prevention of unstable flow in the low-flow region is thus more effectively demonstrated in the numerical analysis compared with the experiments with changes in the angle of the installed IGV at the inlet. According to a previous study^44^, performance and flow stability can be improved using anti-stall fins, similar to the studied IGV component, when such phenomena occur in the inlet sections of fluid machinery. Nevertheless, the occurrence of turning points in the low-flow region and the overall trend at the IGV angles of 25° and 45° are highly similar to the experimental results. Therefore, we proceeded with confidence in the model and grid system used in the numerical analysis.

## Result

### Performance curve and energy-saving scenario

**Fig. 7:**
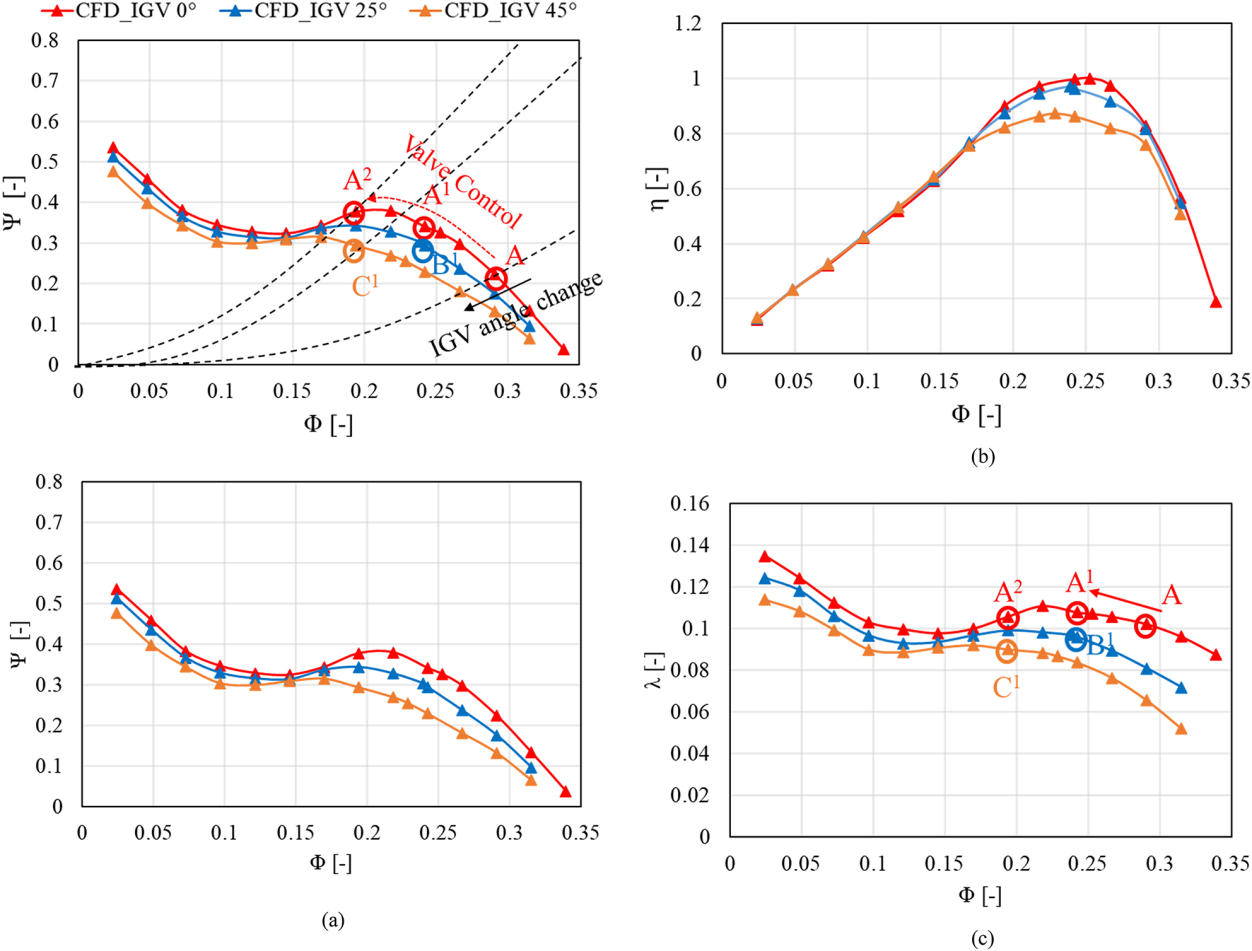
Performance curves of axial flow pump model ( λ : shaft power coefficient). (**a**)  Total head, (**b**)  Total efficiency and (**c**)  Shaft power.

Figure 7 shows the pump performance curves at IGV angles of 0°, 25°, and 45° in the rotation direction. Figure 7(a), (b) and (c) depict the total head, total efficiency, and shaft power performance curves, respectively. In Fig. 7(a), a hypothetical system curve is presented, representing the system curve at the operating flow points A and A^2^ for IGV 0°, and at point C^1^ for an IGV angle of 45°. The hypothetical system curve was depicted to predict the change in the operating flow point as the IGV angle increases from 0° to 25° and 45°. The total head performance curves show that as the IGV angle increases to 25° and 45°, efficiency deteriorates at the same flow coefficient, indicating a reduced operating range. The best efficiency point (BEP) at the IGV angle of 0° is at a flow coefficient of approximately 0.253. As the IGV angle increases to 25° and 45°, the flow coefficient corresponding to the BEP decreases to approximately 0.239 and 0.229, respectively. Furthermore, the absolute value of efficiency corresponding to the BEP also decreases.

During pump operation in the low-flow region, unstable saddle zones occur due to flow rate and pressure fluctuations. The surging phenomenon occurring during saddle zones induces noise and vibration within the pump, adversely affecting its durability and performance characteristics. The changes in the positive slope in the low-flow region are most pronounced at the IGV angle of 0°. The deepest inflection point due to the positive slope is observed at a flow coefficient of approximately 0.145; beyond a flow coefficient of approximately 0.194, the positive slope disappears. Despite the increase in the IGV angle, a positive slope remains in the low-flow region on the overall performance curve, but the slope magnitude decreases compared with that at the IGV angle of 0°. Therefore, although the performance curve shows unstable flow in the low-flow region with an increase in the IGV angle, the region with unstable flow decreases compared with that at the 0° IGV angle. During pump operation with only valve control at a constant IGV angle, operation in the low-flow region has to be avoided due to the occurrence of surging. Through adjustment of the angle of the IGV in the pump, the possibility of pump operation in the low-flow region is confirmed through the reduction of positive slope occurrences. However, although the positive slope in the low-flow region decreases with changes in the IGV angle, it still exists. Conclusions on operability in the low-flow region are difficult to infer based solely on the observed decrease in the positive slope of the performance curve. Therefore, the internal flow phenomena were analyzed to investigate the instabilities occurring within the pump in the low flow rate region as the IGV angle increased to 0°, 25°, and 45°.

Axial flow pumps are mainly used in various sites with frequent load changes, in which case energy is consumed unnecessarily when the operation flow point is changed through valve control (existing method). This can be prevented by establishing an efficient pump operation plan involving the installation of variable-angle IGVs to control the absolute flow angle of the working fluid flowing into the pump. In this study, we predicted the effectiveness of the IGV before installing the variable-angle IGV. Then, we established an efficient, energy-saving pump operation method.

When the load and the target operation flow rate change, at an inlet absolute flow angle of 0°, modifying the operation flow point on the pump power performance curve using valve control increases the axial power consumption. According to Fig. 7(a) and 7(c), as the operating flow rate is changed from point A ($\:{\Phi\:}$ = 0.29) to point A^1^ ($\:{\Phi\:}$ = 0.24) using valve control, the shaft power coefficient increases by approximately 10% from 0.10 to 0.11, respectively. As the operating flow rate is changed from point A ($\:{\Phi\:}$ = 0.29) to point B^1^ ($\:{\Phi\:}$ = 0.24) by adjusting the IGV angle, the shaft power coefficient decreases by approximately 4% from 0.10 to 0.096, respectively. In addition, when the operating flow rate is changed from point A ($\:{\Phi\:}$ = 0.29) to point A^2^ ($\:{\Phi\:}$ = 0.19) through valve control, the shaft power coefficient is 0.11, but when the operating flow rate is changed to point C^1^ ($\:{\Phi\:}$ = 0.19) by changing the IGV angle, the shaft power coefficient is 0.09. When valve control is used to shift the operating flow point from point A to point A^2^, the shaft power coefficient increases by approximately 10%. However, when the IGV angle is changed from 0° to 25°, the shaft power coefficient decreases by approximately 10%. Thus, using the IGV angle change method to change the flow angle in response to load changes significantly reduces the shaft power consumption compared with using valve control.

It can be seen that as the IGV angle increases, the positive slope occurring in the low flow region of the performance curve decreases. In addition, unnecessary shaft power consumption can be reduced by changing the load change response method from valve control (existing method) to IGV angle control (proposed method). Therefore, analyzing the numerical performance curve of the pump at different IGV angles enables the prediction of power consumption according to load changes, so efficient pump operation scenarios can be created.

### Analysis of internal flow field according to IGV angle variations

**Fig. 8:**
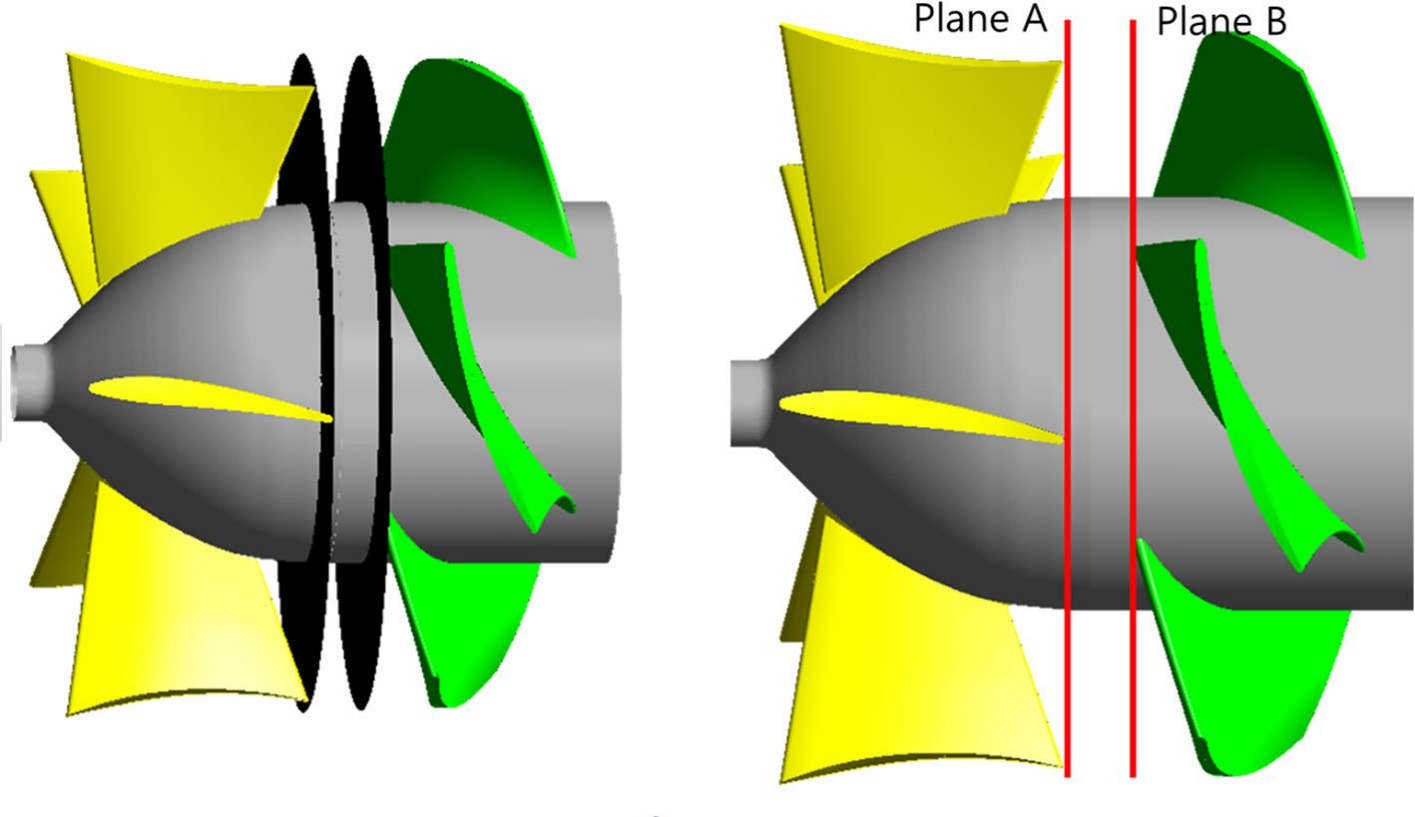
Measurement locations of axial velocity distribution.

In the performance curves in Fig. 7, the positive slope decreases in the low-flow region according to the IGV angle changes. However, assessing flow stability based solely on this decrease in the positive slope is challenging. The variations in the internal flow field with changes in the IGV angle should be analyzed to evaluate flow stability. Therefore, the internal flow in the axial flow pump at various IGV angles was analyzed. Figure 8 illustrates the measurement locations for the analysis of the internal axial velocity distribution. Axial velocity component measurements were conducted at the IGV trailing edge (plane A) and impeller leading edge (plane B). The axial velocity components at plane A and plane B were analyzed to understand the flow behavior when the flow transitions from the IGV to the impeller.

**Fig. 9:**
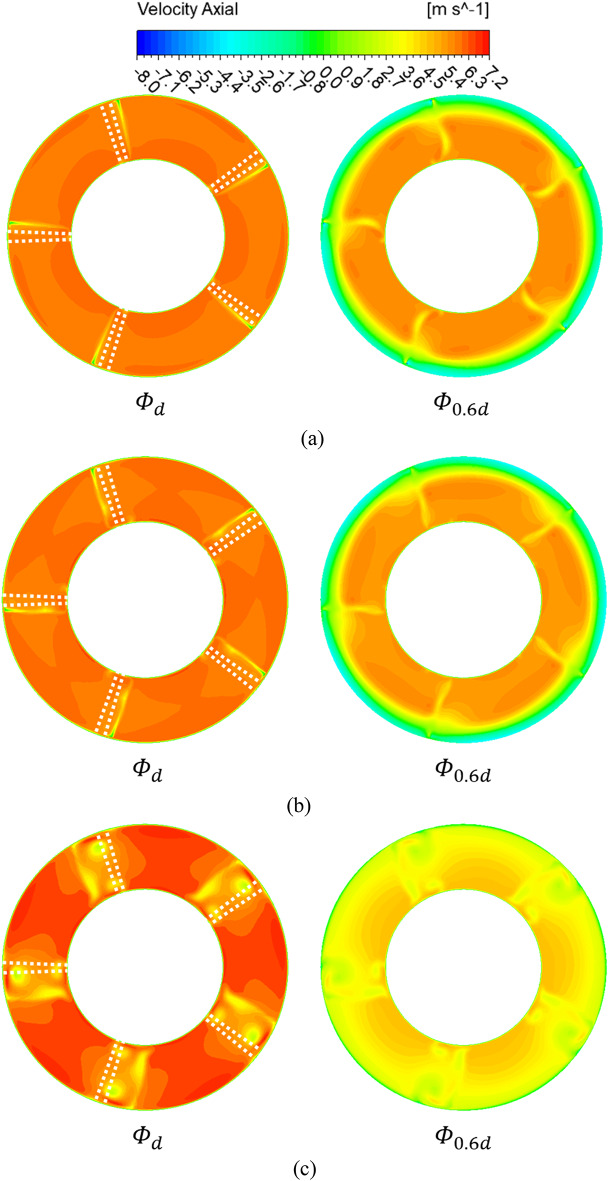
Axial velocity distribution at plane A ($\:{{\Phi\:}}_{d}$: design flow coefficient). (**a**)  IGV angle 0°, (**b**)  IGV angle 25° and (**c**)  IGV angle 45°.

**Fig. 10:**
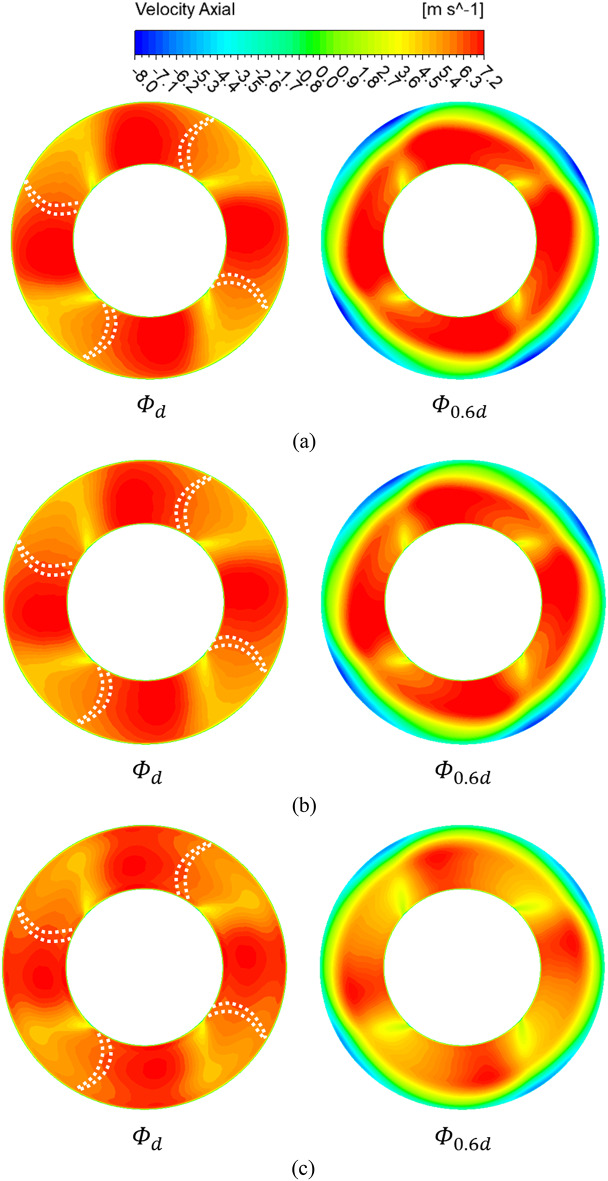
Axial velocity distribution at plane B. (**a**)  IGV angle 0°, (**b**)  IGV angle 25° and (**c**)  IGV angle 45°.

Figure 9 shows the analysis results of the axial velocity components at the IGV shroud leading edge (plane A) at flow coefficient points of 0.242 ($\:{{\Phi\:}}_{d})$ and 0.145 ($\:{{\Phi\:}}_{0.6d})$. The white dashed lines indicate the blade positions at the IGV trailing edge. At the design flow point, no distribution of negative axial velocity components is seen regardless of the IGV angle. This implies that flow separation and backflow do not occur at the design flow point. At the flow coefficient $\:{{\Phi\:}}_{0.6d}$, negative axial velocity components are observed in the shroud region when the IGV angle is 0°. This may be attributed to the IGV tip clearance. However, the absence of negative axial velocity components at the flow coefficient $\:{{\Phi\:}}_{d}$ suggests that the occurrence of such components cannot be solely attributed to the tip clearance. At the IGV angle of 0°, unstable flow phenomena occur near the IGV shroud trailing edge in the low-flow region. When the IGV angle is 25°, negative velocity components are still observed in the shroud region; however, as the angle changes to 45°, the components in the shroud region disappear. Hence, the change in the IGV angle affects the unstable flow phenomenon in the pump. Figure 10 shows the distribution of the axial velocity components at the impeller leading edge (plane B). The white dashed lines mark the impeller blade positions. At the flow coefficient point corresponding to the design flow rate, which is $\:{{\Phi\:}}_{d}$ (similar to that of plane A), no negative axial velocity component distribution is seen regardless of the IGV angle. At the design flow rate point, regardless of the IGV angle, unstable flow does not occur as the flow progresses from the IGV to the impeller. At the flow coefficient point corresponding to the low-flow condition, which is $\:{{\Phi\:}}_{0.6d}$, despite the change in the IGV angle from 0° to 45°, a negative axial component distribution is observed. However, at the IGV angle of 25°, the intensity of negative axial velocity components is lower than that at 0°. Furthermore, at the IGV angle of 45°, the distribution of negative axial velocity components significantly decreases compared with those at 0° and 25°. Hence, changing the IGV angle affects the angle of the flow entering the impeller, which in turn affects the unstable internal flow phenomena.

**Fig. 11:**
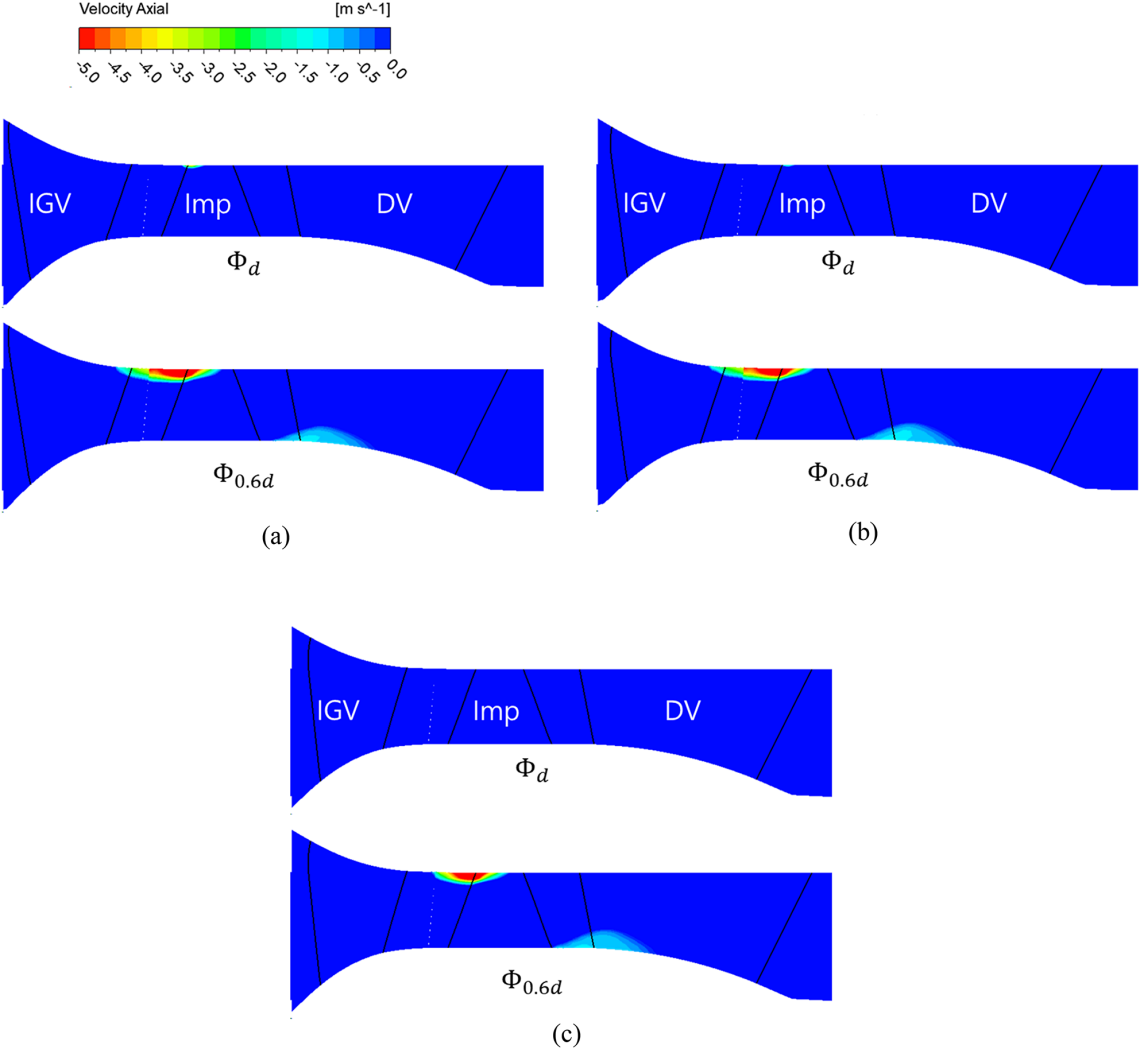
Axial velocity distribution in IGV model meridional section (DV: diffuser vane). (**a**)  IGV angle 0°, (**b**) IGV angle 25° and (**c**)  IGV angle 45°.

Figure 11 shows a meridional view of the distribution of axial velocity components at $\:{{\Phi\:}}_{d}$ and $\:{{\Phi\:}}_{0.6d}$. At $\:{{\Phi\:}}_{d}$, a weak presence of a negative axial velocity component distribution is detected near the impeller shroud at the 0° IGV angle. However, these components near the impeller shroud cannot be easily considered to have a significant influence on performance characteristics due to their narrow distribution range. Additionally, it can be noted this distribution disappears as the IGV angle changes to 45°. At $\:{{\Phi\:}}_{0.6d}$, at the 0° IGV angle, a negative axial velocity component distribution appears from around the IGV shroud trailing edge to the impeller shroud area. Hence, at $\:{{\Phi\:}}_{0.6d}$, backflow occurs from the IGV trailing edge to the impeller, affecting pump performance. Backflow negatively impacts the flow entering the impeller, influencing the pump performance characteristics adversely. This distribution of negative axial velocity components decreases in magnitude at the IGV angle of 25° and significantly diminishes at 45°. Thus, changes in the IGV angle affect the angle of incidence of the flow entering the impeller. In turn, changes in the angle of incidence due to IGV angle adjustment affect the backflow phenomena occurring inside the pump.

**Fig. 12:**
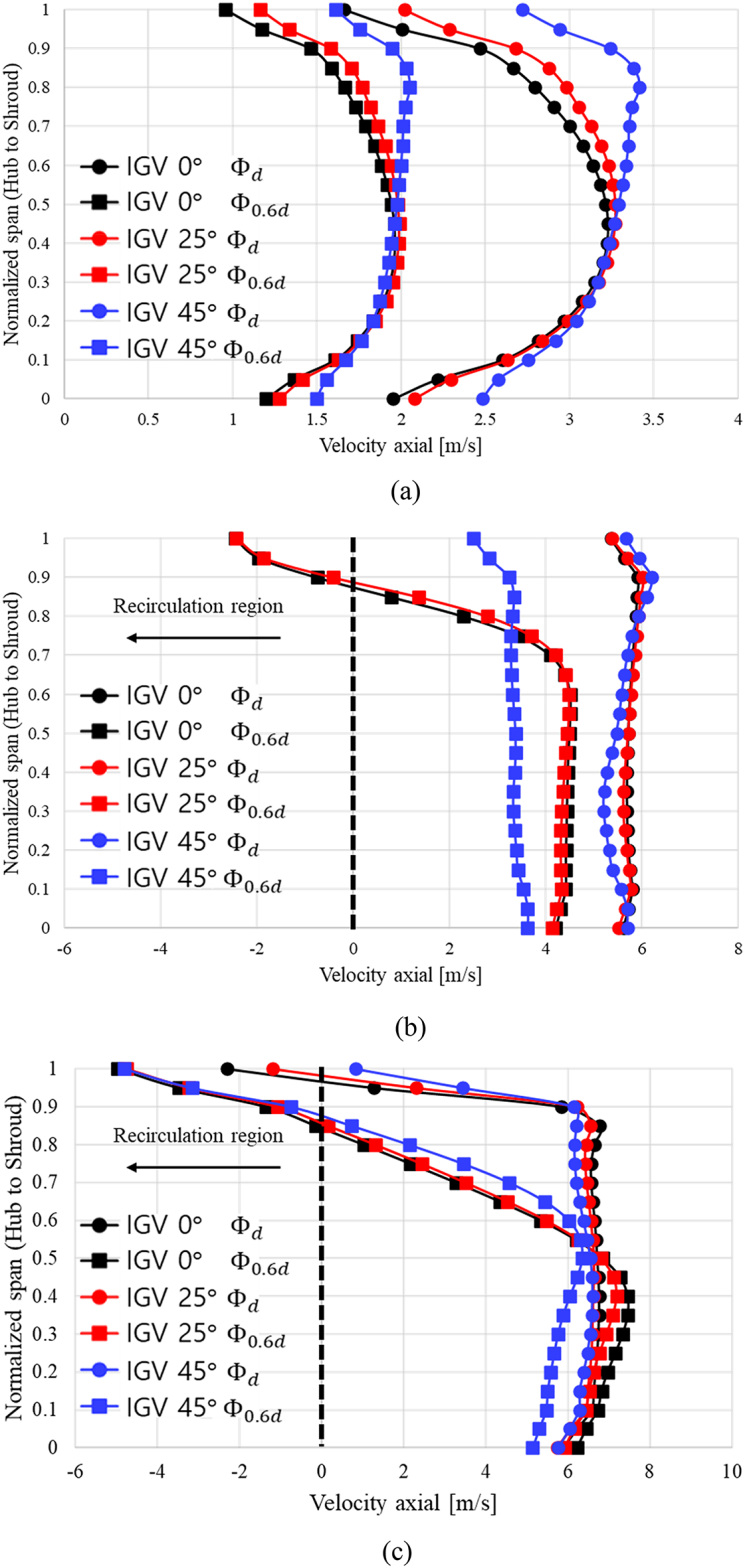
Axial velocity distribution from hub to shroud span according to measurement positions.(**a**) Position of IGV leading edge, (**b**)  Position of IGV trailing edge and (**c**)  Position of impeller leading edge.

Figure 12 graphically depicts the distributions of axial velocity components at the IGV leading edge (Fig. 12(a)), IGV trailing edge (Fig. 12(b)), and impeller leading edge (Fig. 12(c)) at $\:{{\Phi\:}}_{d}$ and $\:{{\Phi\:}}_{0.6d}$. The x axis represents the axial velocity component at each position, and the y axis represents the distance from the hub to the shroud at each location. At the IGV leading edge, regardless of the IGV angle, no distribution of negative axial velocity components is seen at $\:{{\Phi\:}}_{d}$ and $\:{{\Phi\:}}_{0.6d}$. Therefore, regardless of changes in the IGV angle and flow coefficient, the flow entering from the inlet does not undergo backflow. At the IGV trailing edge, regardless of changes in the IGV angle, no distribution of negative axial velocity components is observed at $\:{{\Phi\:}}_{d}$. At $\:{{\Phi\:}}_{0.6d}$, negative axial velocity components start to appear from approximately 0.85 span at the IGV angle of 0°. A similar trend occurs at the IGV angle of 25°. As shown in Fig. 11, flow separation occurs at the IGV trailing edge at approximately 0.85 span onward. Moreover, at $\:{{\Phi\:}}_{0.6d}$ and IGV angles of 0° and 25°, unstable backflow occurs from the IGV trailing edge. The negative axial velocity components observed at the IGV angles of 0° and 25° disappear as the angle changes to 45°. At $\:{{\Phi\:}}_{d}$ at the impeller leading edge, negative axial velocity components appear at 0.95 and 0.97 span at the IGV angles of 0° and 25°, respectively. As observed in Fig.  11(a) and (b), at $\:{{\Phi\:}}_{d}$, a negative axial velocity component distribution is identified in the impeller shroud region despite it being the design flow point. Based on Fig. 11(a), (b), and 12(c), the distribution of negative axial velocity components in the impeller shroud region at $\:{{\Phi\:}}_{d}$ is attributed to the IGV tip clearance. At the IGV angle of 45°, no distribution of negative axial velocity components is seen in the impeller shroud region; likewise, no such components are observed in Fig. 12(c). This confirms that changes in the IGV angle affect the incidence angle of the flow entering the impeller. At $\:{{\Phi\:}}_{0.6d}$, negative axial velocity components are observed at the IGV angles of 0°, 25°, and 45°. These components appear from approximately 0.85, 0.87, and 0.89 span, respectively.

Despite the adjustment of the IGV angle to modify the angle of flow incidence at low flow rates, negative axial velocity components are still observed. However, the position where these components occur shifts closer to the shroud span side as the IGV angle changes. Hence, the variation in the IGV angle affects the unstable flow in the pump. The internal flow phenomena were analyzed with respect to changes in the IGV angle at $\:{{\Phi\:}}_{d}$ and $\:{{\Phi\:}}_{0.6d}$. The results confirm that the IGV angle influences the internal unstable flow phenomena. However, it is difficult to determine accurately whether the pump operates stably under low-flow conditions based solely on the analysis of the flow phenomena within the pump. For accurate analysis, internal flow stability was assessed.

### Analysis of fast Fourier transform (FFT) results

At $\:{{\Phi\:}}_{d}$ and $\:{{\Phi\:}}_{0.6d}$, FFT analysis using pressure pulsations was performed to evaluate the internal flow stability as the IGV angle changes to 0°, 25°, and 45°. The blade passage frequency (BPF) in this study is 170.67 Hz and expressed as Eq. (5).

5 $$\:BPF\:\left({f}_{n}\right)=\:\frac{ZN}{60}$$

In Eq. (5), $\:Z$ and $\:N$ are the numbers of blades and revolutions per minute, respectively.

**Fig. 13:**
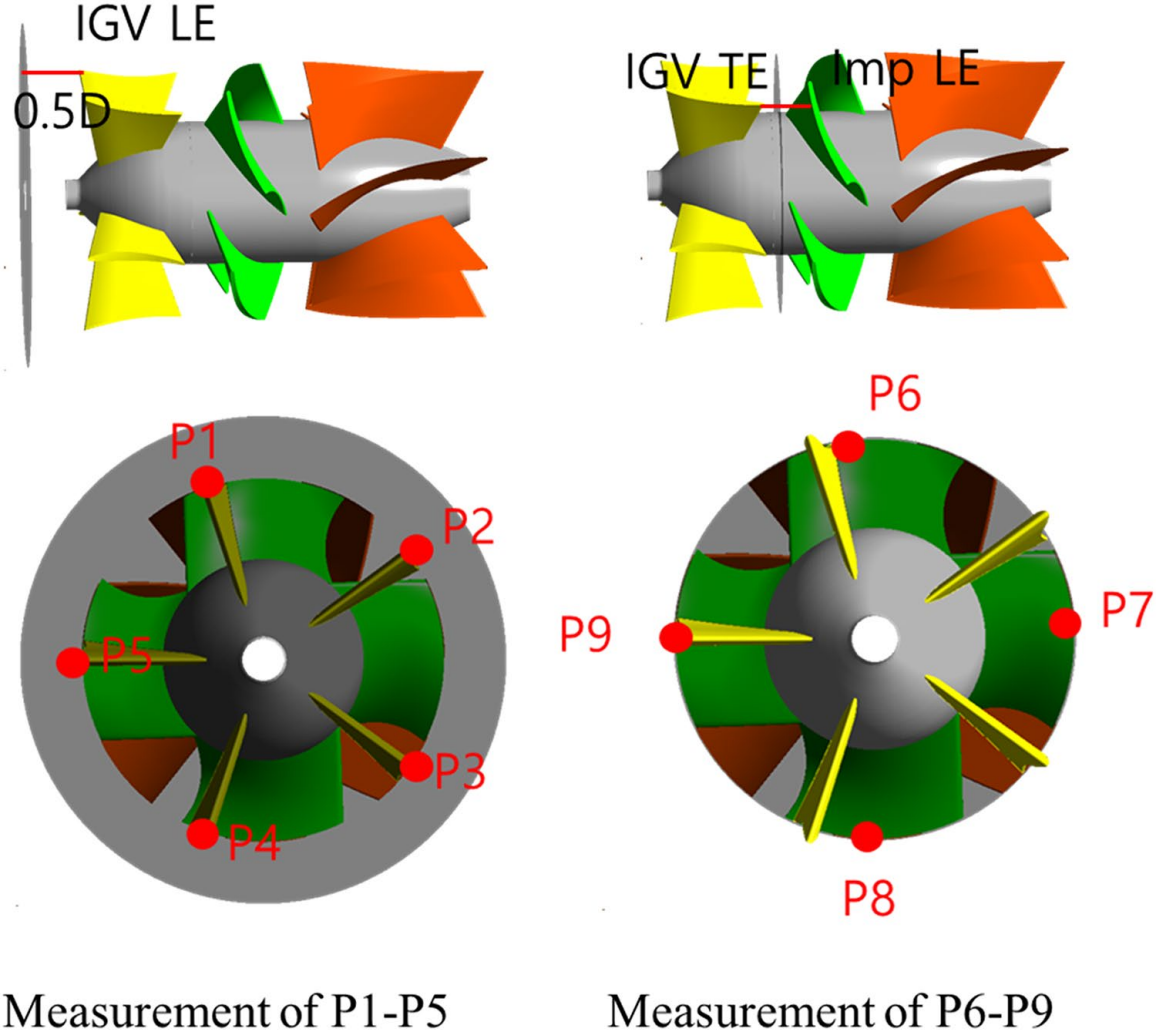
Locations of pressure pulsation measurements (LE: leading edge; TE: trailing edge).

Figure 13 shows the measurement locations within the pump for pressure pulsation analysis. P1–P5 were measured at positions located 0.5$\:D$ toward the inlet side from the IGV leading edge. Measurements were obtained at each blade shroud leading edge of the IGV. P6–P9 were measured at intermediate points between each impeller shroud leading edge and the IGV trailing edge. We also measured pressure pulsations based on the pressure difference between the inlet and outlet sections. This allowed us to assess flow stability at $\:{{\Phi\:}}_{d}$ and $\:{{\Phi\:}}_{0.6d}$ and analyze the influence of the IGV angle on flow stability.

**Fig. 14:**
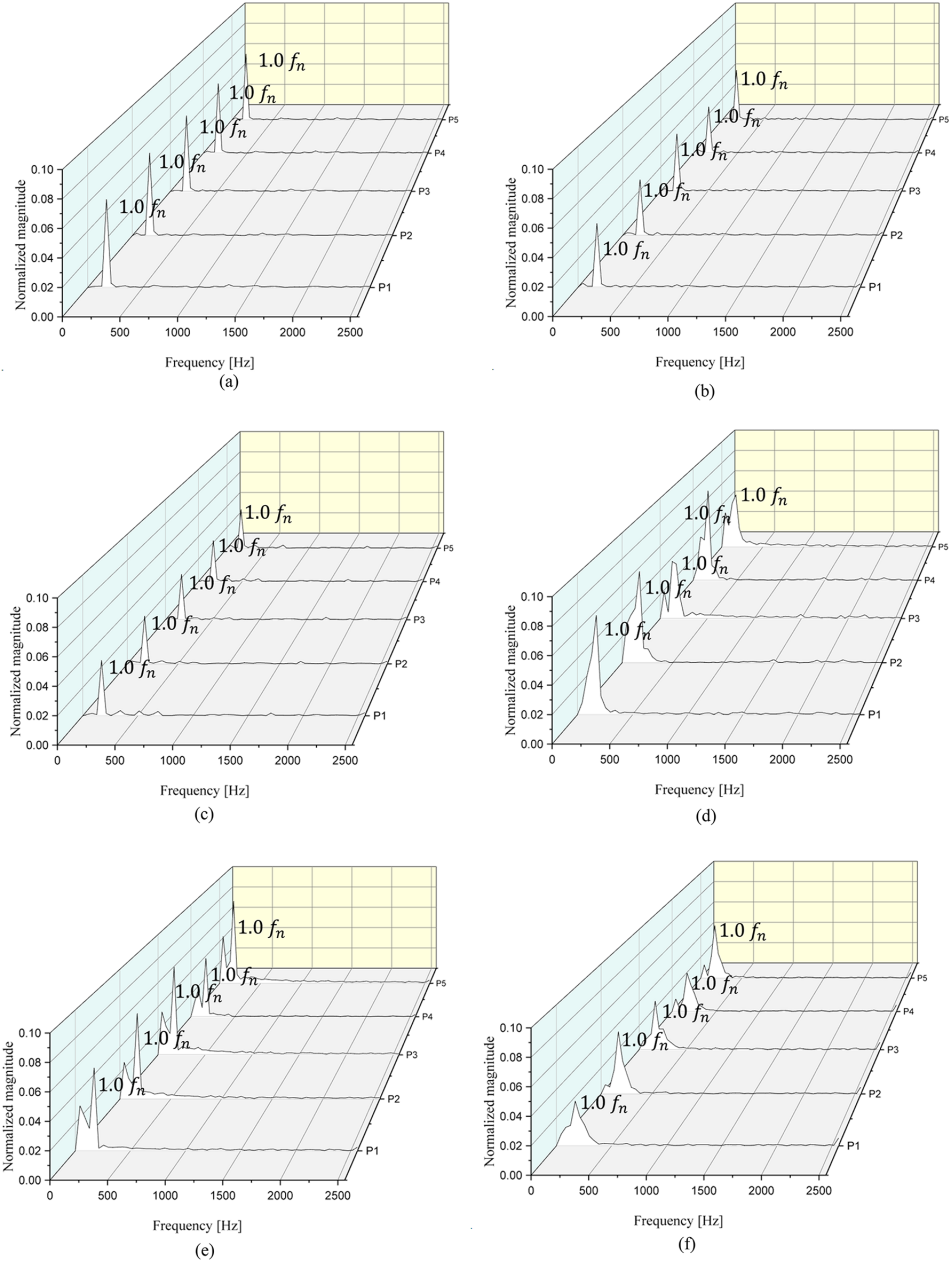
FFT results from P1 to P5. (**a**) IGV angle 0°, $\:{{\Phi\:}}_{d}$ (**b**) IGV angle 25°, $\:{{\Phi\:}}_{d}$, (**c**) IGV angle 45°, $\:{{\Phi\:}}_{d}$, (**d**) IGV angle 0°, $\:{{\Phi\:}}_{0.6d}$, (**e**) IGV angle 25°, $\:{{\Phi\:}}_{0.6d}$ and (**f**) IGV angle 45°, $\:{{\Phi\:}}_{0.6d}$. .

**Fig. 15:**
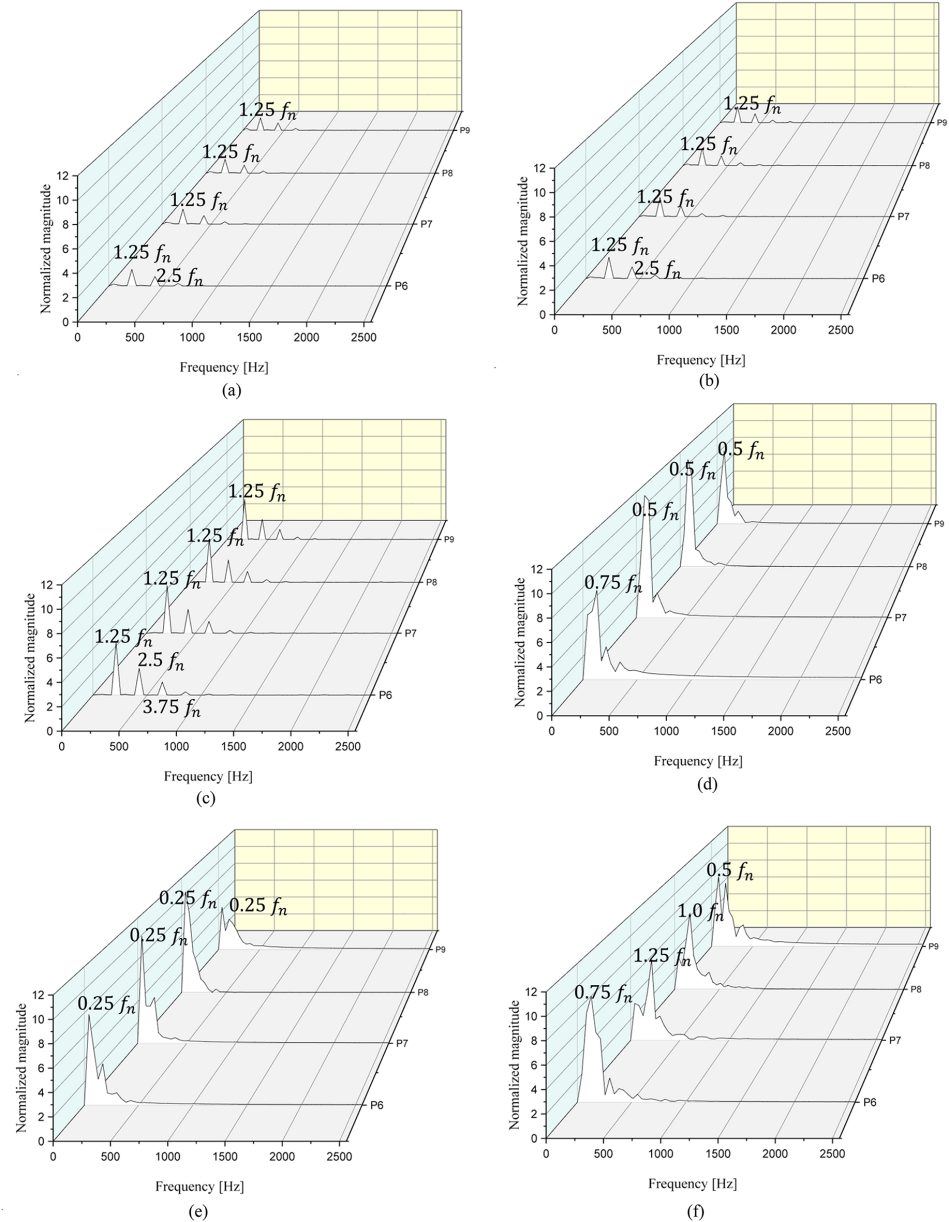
FFT results from P6 to P9. (**a**) IGV angle 0°, $\:{{\Phi\:}}_{d}$,(**b**) IGV angle 25°, $\:{{\Phi\:}}_{d}$,(**c**) IGV angle 45°, $\:{{\Phi\:}}_{d}$,(**d**) IGV angle 0°, $\:{{\Phi\:}}_{0.6d}$,(e) IGV angle 25°, $\:{{\Phi\:}}_{0.6d}$ and (**f**) IGV 45°, $\:{{\Phi\:}}_{0.6d}$.

**Fig. 16:**
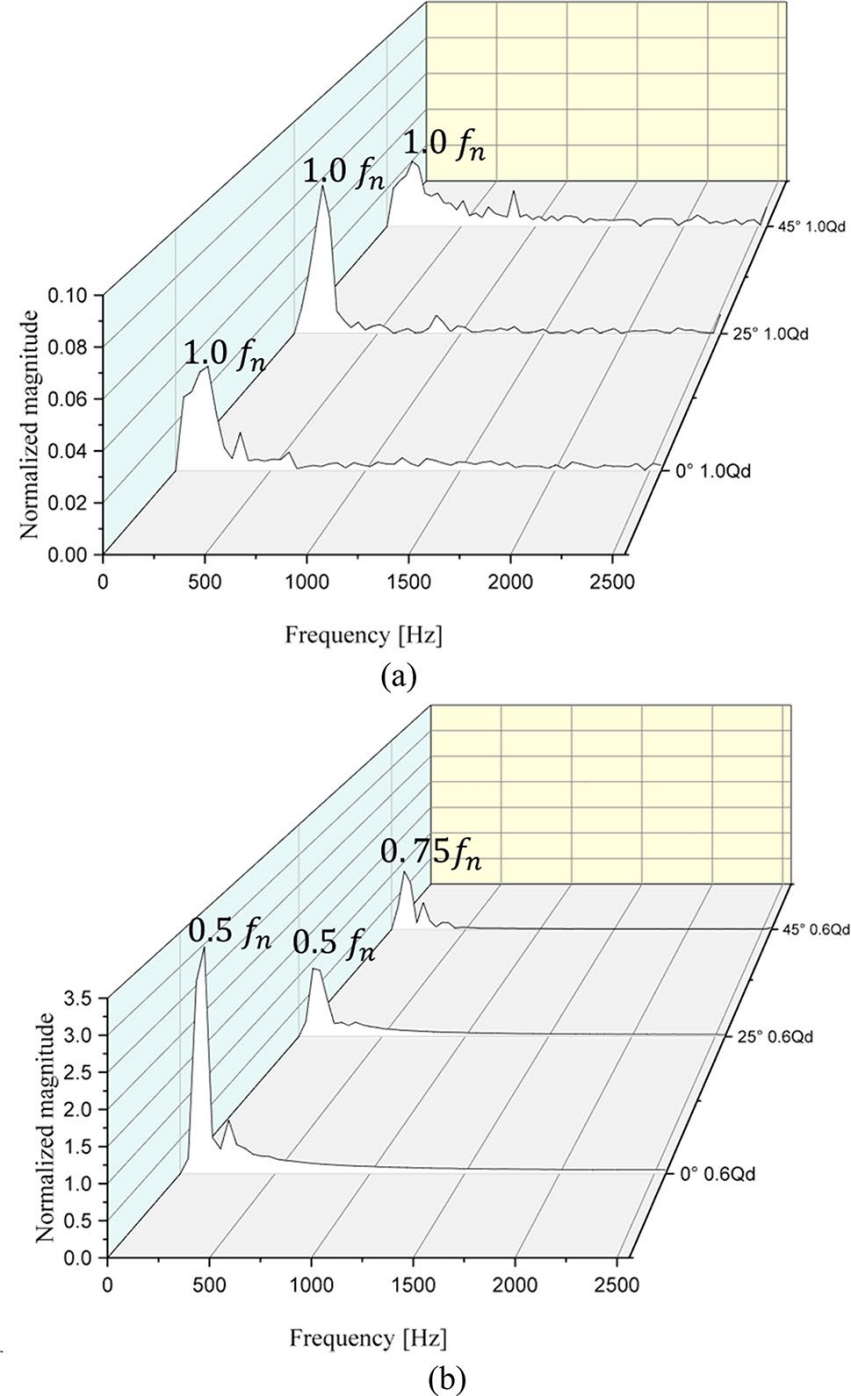
FFT results for total pressure fluctuation at each IGV angle. (**a**) $\:{{\Phi\:}}_{d}$ and (**b**) $\:{{\Phi\:}}_{0.6d}$.

Figures 14 and 15, and 16 show the FFT results corresponding to the different IGV angles at $\:{{\Phi\:}}_{d}$ and $\:{{\Phi\:}}_{0.6d}$. The x axis indicates frequency, and the y axis is the normalized magnitude of pressure fluctuation. The magnitude of pressure fluctuations has been normalized to the pressure value corresponding to the total head at the design flow point with the IGV angle of 0°. A normalized frequency of $\:{1.0f}_{n}$ corresponds to 170.67 Hz, which is the BPF. At P1 to P5, the highest peak occurs at $\:{1.0f}_{n}$ at both $\:{{\Phi\:}}_{d}$ and $\:{{\Phi\:}}_{0.6d}$. Furthermore, the largest peak is observed at $\:{1.0f}_{n}$ at all IGV angles. Regardless of the variations in the flow coefficient and IGV angle, at a point located 0.5$\:D$ toward the inlet side from the IGV leading edge, a peak corresponding to the BPF appears. Therefore, no unstable flow components occur at P1–P5. This indicates that the flow entering from the inlet is stable regardless of the flow coefficient.

The FFT results at P6–P9, measured at the middle position between the IGV trailing edge and the impeller leading edge, are presented in Fig. 15. At $\:{{\Phi\:}}_{d}$, the frequency distribution is similar across all IGV angles. The peak frequency from P6 to P9 is at $\:{1.25f}_{n}$ regardless of the IGV angle. Additionally, secondary and tertiary peak frequencies are observed at $\:{2.5f}_{n}$ and $\:{3.75f}_{n}$, respectively, indicating periodic occurrence at intervals of $\:{1.25f}_{n}$. Despite being at the design flow coefficient, the peak frequency occurs after the BPF because the measurements were obtained at the same location as the IGV trailing edge. As depicted in Figs. 11 and 12, negative axial velocity components are present in the shroud region between the IGV and the impeller despite being at the design flow point, $\:{{\Phi\:}}_{d}$. This location experiences unstable flow phenomena due to the presence of the IGV tip clearance. Therefore, the peak frequency is observed after the BPF due to the tip clearance. However, the magnitude of pressure fluctuation in the low-flow region is significantly smaller in comparison. Additionally, the secondary and tertiary peak frequencies are periodically observed after the primary peak frequency; such a pattern of pressure fluctuations have a minimal impact on flow instability during pump operation. Moreover, the magnitude increases at P6–P9 at $\:{{\Phi\:}}_{d}\:$as the IGV angle changes from 0° to 45°. At $\:{{\Phi\:}}_{0.6d}$, when the IGV angle is 0°, the peak points from P6 to P9 are observed at $\:{0.75f}_{n}$, $\:{0.5f}_{n}$, $\:{0.5f}_{n}$, and $\:{0.5f}_{n}$. The peak points observed after the primary peak point do not exhibit periodicity. The largest magnitude observed is approximately 11. Thus, at the IGV angle of 0°, the flow entering from the impeller is unstable. At the IGV angle of 25°, the peak points from P6 to P9 occur consistently at $\:{0.25f}_{n}$. The largest magnitude is approximately 10 (at P8). At the IGV angle of 45°, the peak points from P6 to P9 occur at $\:{0.75f}_{n}$, $\:{1.25f}_{n}$, $\:{1.0f}_{n}$, and $\:{0.5f}_{n}$. The largest magnitude is approximately 8 (at P6). This confirms the presence of unstable flow phenomena in the low-flow region at $\:{{\Phi\:}}_{0.6d}$ regardless of the change in the IGV angle from 0° to 45°. As the IGV angle changes from 0° to 25° and 45°, the largest magnitudes decrease to 11, 10, and 8, respectively. Additionally, at the IGV angle of 45°, a peak point corresponding to the BPF at $\:{1.0f}_{n}$ is observed at P8.

Figure 16 shows the FFT results for the pressure fluctuations derived from the total pressure difference in the inlet and outlet sections at $\:{{\Phi\:}}_{d}$ and $\:{{\Phi\:}}_{0.6d}$. At $\:{{\Phi\:}}_{d}$, peak points are observed at the BPF regardless of the IGV angle. The largest magnitudes are small, approximately 0.04, 0.06, and 0.02. Thus, the internal flow at $\:{{\Phi\:}}_{d}$ is stable overall. At $\:{{\Phi\:}}_{0.6d}$, peak points are observed at $\:{0.5f}_{n}$, $\:{0.5f}_{n}$, and $\:{0.75f}_{n}$ at the IGV angles of 0°, 25°, and 45°, respectively. The largest magnitudes are approximately 3.0, 1.0, and 0.9, respectively. Hence, at $\:{{\Phi\:}}_{0.6d}$, the magnitude is significantly larger at the IGV angle of 0°, indicating a highly unstable internal flow. Peak values are also observed at frequencies before the BPF at the IGV angles of 25° and 45°, inducing unstable flow patterns. However, the magnitude significantly decreases as the IGV angle changes from 0° to 25° and 45°. The FFT measurement results at P6–P9 in the low-flow region also confirm the occurrence of unstable flow regardless of the IGV angle; however, the magnitude significantly decreases as the IGV angle changes to 45°. This confirms the presence of unstable flow patterns in the low-flow region regardless of the IGV angle. However, as the IGV angle changes to 45°, the internal flow significantly stabilizes relative to that at the IGV angle of 0°. Therefore, in the low-flow region, changing the IGV angle results in a more stable pump operation compared with pump operation at the IGV angle of 0°.

### Application of pump operation scenarios

**Fig. 17:**
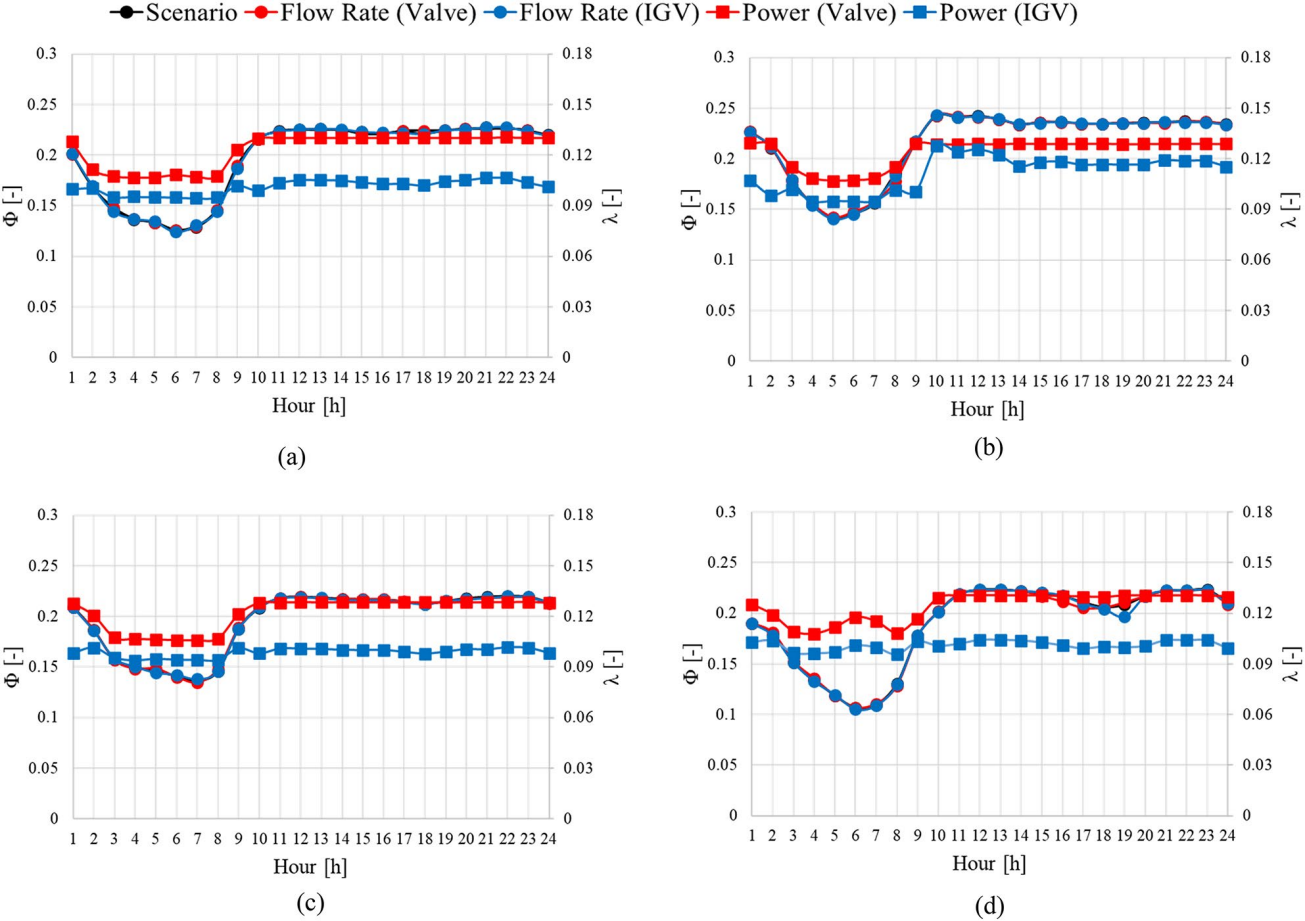
Predicted pump operation scenarios for sewage treatment plant S. (**a**)  Spring, (**b**)  Summer , (**c**)  Fall and (**d**)  Winter.

**Fig. 18:**
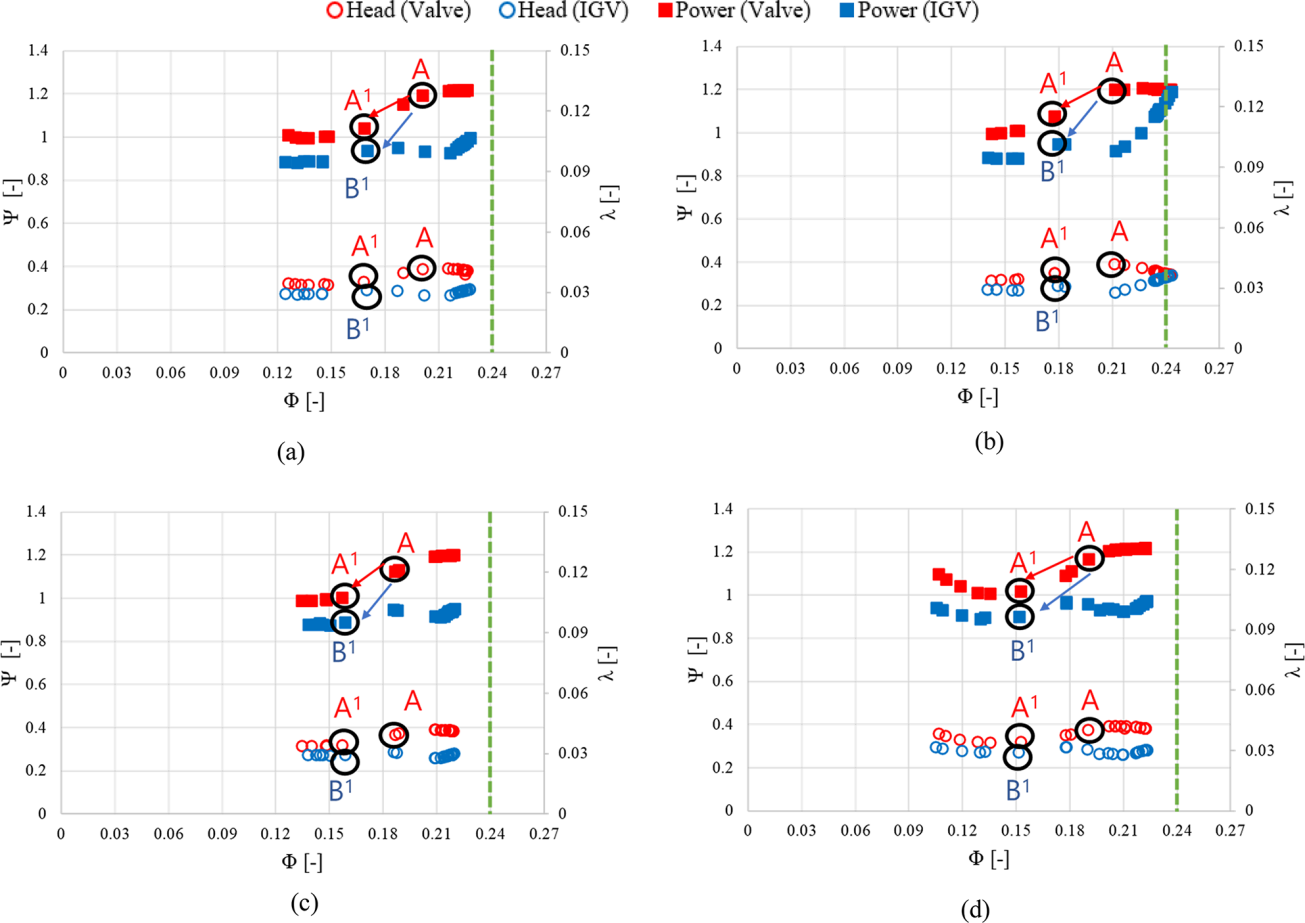
Predicted pump performance for sewage treatment plant S.  (**a**)  Spring, (**b**)  Summer , (**c**)  Fall and (**d**)  Winter.

**Fig. 19:**
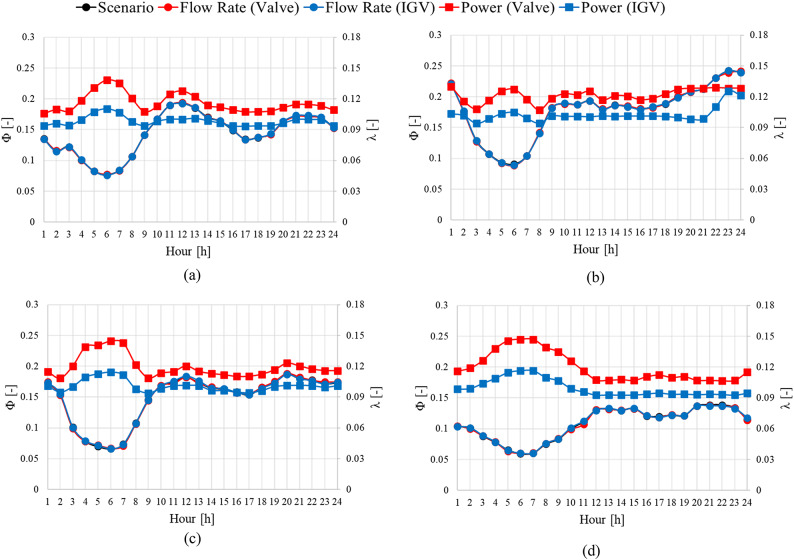
Predicted pump operation scenarios for sewage treatment plant H. (**a**)  Spring, (**b**)  Summer , (**c**)  Fall and (**d**)  Winter.

**Fig. 20:**
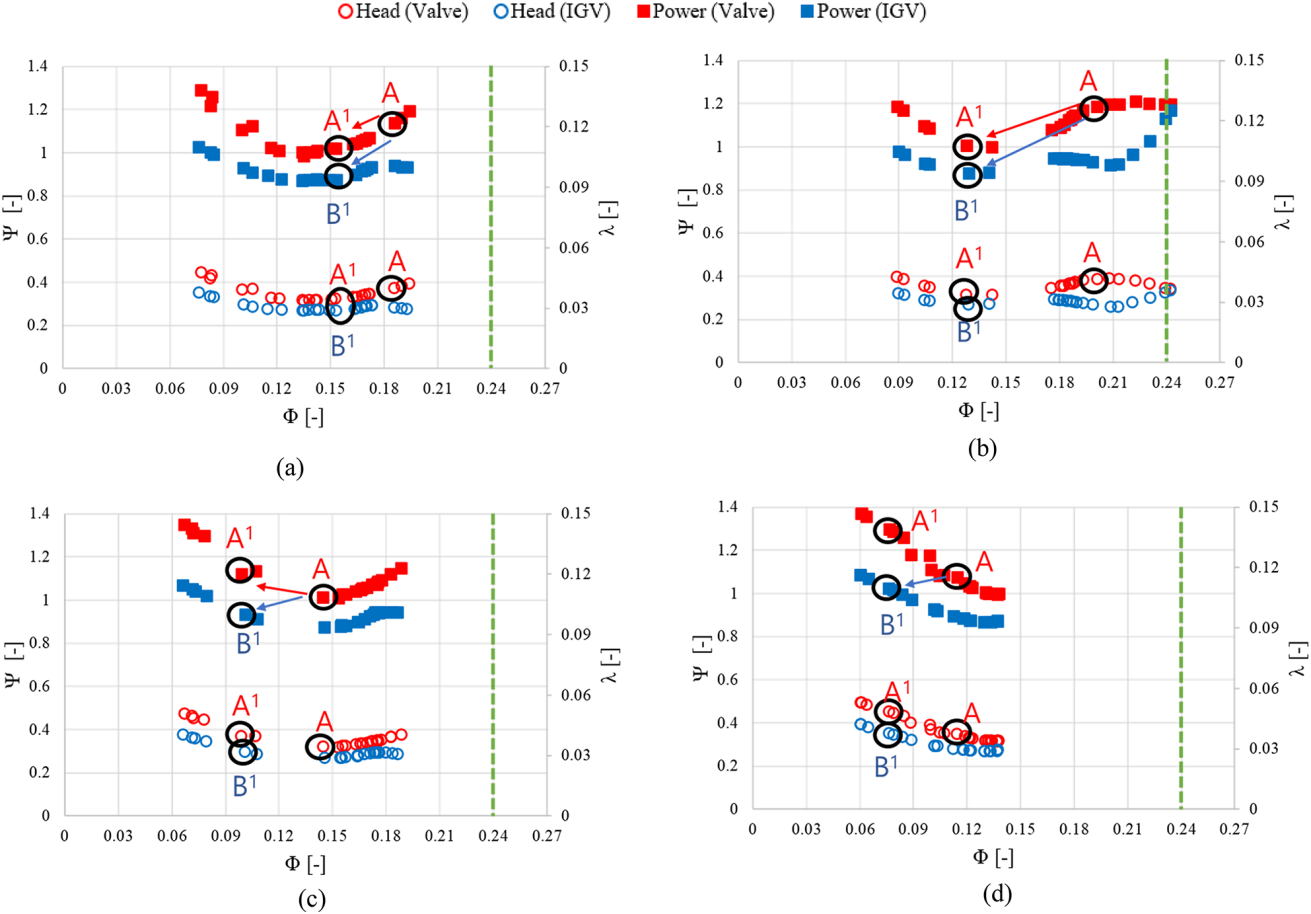
Predicted pump performance for sewage treatment plant H. (**a**) Spring, (**b**) Summer , (**c**) Fall and (**d**)  Winter.

Experiments were conducted using the model in this study to validate the abovementioned energy-saving methods. The experimental devices are shown in Fig. 3, and the experiments were conducted at the KITECH. The data used in the experiments were based on the actual operational data of sewage treatment plants S and H in Korea and consisted of specific monthly average time data. We selected representative months for each season: April (spring), July (summer), October (fall), and January (winter). Figures 17 and 18 depict the operating scenarios predicted based on load-change control methods and the total head and shaft power curves at various flow rates variation using the monthly average operational data from sewage treatment plant S. Figures 19 and 20 show the predicted operating scenarios and performance curve experimental results obtained based on actual operational data from sewage treatment plant H. Table 3 presents the energy consumption based on the method of responding to load changes during pump operation. Based on the actual operational data of both treatment plants, when the pumps were operated using valve control and IGV angle change, the maximum error relative to the actual data was less than 2%. Therefore, the pumps were operated similarly to the actual operational data. The experimental results showed that operating the pumps of sewage treatment plant S using the IGV angle adjustment method to respond to load fluctuations resulted in an average reduction of approximately 0.49 in the coefficient of the shaft power consumption compared with that under valve control. Additionally, energy consumption was reduced on average by approximately 15.63%. Among the seasons, summer showed the highest consumption of shaft power, whereas winter exhibited the lowest. Therefore, the difference in energy saving was the least in summer (approximately 10.20%) and the highest in winter (18.69%). The performance curves in Fig. 18 are similar to the ones in Fig. 7. When load changes occur at a specific flow coefficient point A, the operating flow point is changed to points A^1^ and B^1^ by adjusting the valve and IGV angle, respectively. In this case, the coefficients of the shaft power consumed when responding to load changes in spring, summer, fall, and winter through IGV angle adjustment were lower compared with those under valve control. When IGV angle adjustment was used instead of valve control, the total head and shaft power coefficients decreased across the entire flow range. Hence, the IGV angle adjustment method is effective in reducing shaft power consumption not only in the high-flow region but also in the low-flow region. However, when the inflow flow rate ($\:{\Phi\:}$ = 0.24) exceeded a certain threshold, such as during the summer, applying the IGV angle adjustment method did not reduce the shaft power consumption.

**Table 3:** Application results of pump operation scenarios.

Season	Monthly average energy savings	Sewage treatment plant	
		S	H
Spring	Shaft coefficient (-)	0.53	0.43
	Energy saving rate (%)	17.93	15.29
Summer	Shaft coefficient (-)	0.31	0.46
	Energy saving rate (%)	10.20	15.60
Fall	Shaft coefficient (-)	0.57	0.45
	Energy saving rate (%)	15.70	15.70
Winter	Shaft coefficient (-)	0.56	0.49
	Energy saving rate (%)	18.69	16.97

As for sewage treatment plant H, the IGV angle adjustment method resulted in an average reduction of approximately 0.46 in shaft power consumption compared with the valve method. The average energy consumption was approximately 15.89% lower under the IGV angle change method than under valve control. Unlike the case of sewage treatment plant S, the shaft power consumption of sewage treatment plant H was similar in spring and summer. Additionally, the energy saving in winter (approximately 16.97%) was the highest among all seasons. Similar to the results obtained using the data of sewage treatment plant S, those acquired using the data of sewage treatment plant H confirmed that the shaft power consumption remained similar beyond the flow rate of $\:{\Phi\:}$ = 0.24 regardless of the method used (Fig. 20). Furthermore, in the case of sewage treatment plant H, the pump operated in the low-flow region throughout all seasons, unlike that of sewage treatment plant S. During pump operation using the IGV angle adjustment method in the low-flow region at $\:{\Phi\:}$ = 0.1 or less, the shaft power consumption significantly decreased. Given a load change at a specific flow coefficient A, adjusting the IGV angle to change the operating flow point to B^1^ reduced the shaft power consumption coefficient compared to changing the operating flow point to A^1^ via valve control. This result was similar to that of the operating scenario for sewage treatment plant S.

The pumps were operated using actual sewage treatment plant data for the representative month of each season. Pump performance was compared between the valve control and IGV angle adjustment methods of adapting to load fluctuations. The IGV angle adjustment method reduced energy consumption by approximately 15% on average compared with the valve adjustment method. According to the predicted operating scenarios based on performance curves, the IGV angle adjustment method effectively decreased the shaft power consumption relative to the valve control method in response to load changes. Therefore, using the IGV angle adjustment method instead of the conventional method can reduce energy consumption while effectively operating axial flow pumps. This suggests that efficient operation can be achieved based on energy consumption predictions and varying efficiencies between seasons; numerical analyses should be conducted to predict pump operating scenarios before installing these pumps on-site and then manufacturing and applying them accordingly.

## Conclusions

This study aims to evaluate flow stability in the low-flow region and predict energy saving during actual pump operation by adjusting the IGV angle inside the axial flow pump. Internal flow phenomena and FFT results were analyzed to examine flow stability according to IGV angle changes. Actual sewage treatment plant data were applied to operate pumps for predicting energy savings. Energy consumption values during the use of valve control and IGV angle adjustment in response to load changes were then compared.

First, analysis of pump performance characteristics at IGV angles of 0°, 25°, and 45° revealed that as the IGV angle increased, the operable flow coefficient range of the pump decreased. The changes in the positive slope in the low-flow region were most pronounced at the IGV angle of 0°, gradually decreasing as the angle increased to 45°. Performance curve characteristics were analyzed to predict pump operation scenarios through valve control and IGV angle adjustment. Findings showed that the energy consumption under IGV angle adjustment, indicated by the shaft power coefficient, decreased by approximately 10% compared with that under valve control.

Second, analysis was conducted on the internal flow phenomena at $\:{{\Phi\:}}_{d}$ and $\:{{\Phi\:}}_{0.6d}$ according to different IGV angles. At $\:{{\Phi\:}}_{d}$, no negative axial velocity component was observed regardless of the IGV angle. However, at $\:{{\Phi\:}}_{0.6d}$, negative axial velocity components persisted despite the IGV angle changes. Nonetheless, the area of distribution of these components significantly decreased as the IGV angle changed from 0° to 25° and 45°.

Third, FFT results were analyzed to evaluate flow stability at various IGV angles at $\:{{\Phi\:}}_{d}$ and $\:{{\Phi\:}}_{0.6d}$. At $\:{{\Phi\:}}_{d}$, the flow remained stable regardless of the IGV angle. However, at $\:{{\Phi\:}}_{0.6d}$, unstable flow patterns appeared at the IGV angles of 0°, 25°, and 45°. In particular, at the IGV angle of 0°, the magnitude of the frequencies inducing the unstable flow patterns was significantly high. Nevertheless, as the IGV angle transitioned to 25° and 45°, the frequency magnitude decreased notably. Therefore, under IGV angle adjustment, stable operation could be achieved at $\:{{\Phi\:}}_{0.6d}$ relative to that at the IGV angle of 0°.

Fourth, experiments were conducted using real operational data from two sewage treatment plants in Korea. These experiments involved applying valve control and IGV angle adjustment in response to actual load changes. Operating using IGV angle adjustment reduced the shaft power coefficient compared with that under valve control. Changing the IGV angle led to an average reduction in energy consumption of approximately 15%.

## Data Availability

The data that support the findings of this study are available from the corresponding author upon request.

## List of symbols

$$\:A$$: Area (m^2^)
$$\:c$$: Absolute velocity (m/s)
$\:{C}_{m2}$: Meridional component of absolute velocity at impeller outlet (m/s)
$$\:D$$: Diameter
$$\:g$$: Gravitational acceleration (m/s^2^)
$$\:H$$: Total head (m)
$$\:L$$: Shaft power (W)
$\:l$: Length of the recirculation region from the impeller shroud tip
$$\:{N}_{s}$$: Specific speed
$\:Q$: Flow rate (CMM)
$$\:u$$: Rotational velocity (m/s)
$\:{u}_{2}$: Impeller outlet rotational velocity (m/s)
$$\:w$$: Relative velocity (m/s)
$\:\beta\:$: Absolute flow angle (°)
$$\:\rho\:$$: Density (kg/m^3^)
$\:\omega\:$: Angular velocity (rad/s)
$$\:{\Phi\:}$$: Flow coefficient
$\:{{\Phi\:}}_{d}$: Design flow coefficient
$\:\psi\:$: Total head coefficient
$\:\eta\:$: Normalized total efficiency
$\:\lambda\:$: Shaft power coefficient
$\:\theta\:$: Inlet flow angle (°)
